# Supplementary material for: TrimNN: characterizing cellular community motifs for studying multicellular topological organization in complex tissues
Source: Nat Commun. 2025 Aug 19;16:7737. doi: 10.1038/s41467-025-63141-7 (PMC12365081; doi:10.1038/s41467-025-63141-7)
Supplement: Supplementary file 1 — Supplementary Information [file 41467_2025_63141_MOESM1_ESM.pdf]

Supplementary Figure

	Top-down	Bottom-up		
**Approach**	Clustering	Enumeration	Permutation	TrimNN
**Interpretability**	Low	High		
**Generalizability**	Low	High		
**Designated sizes**	No	Yes		
**Size 1-3 Accuracy**	NA	Ground truth	Low	High
**Size 1-3 Speed**	Very Fast	Fast	Fast	Fast
**Size 4 and up Accuracy**	NA	Not feasible	Low	High
**Size 4 and up Speed**	Very Fast	Not feasible	Fast	Fast

Supplementary Fig.1 Comparison of performance between top-down and bottom-up approaches**.**

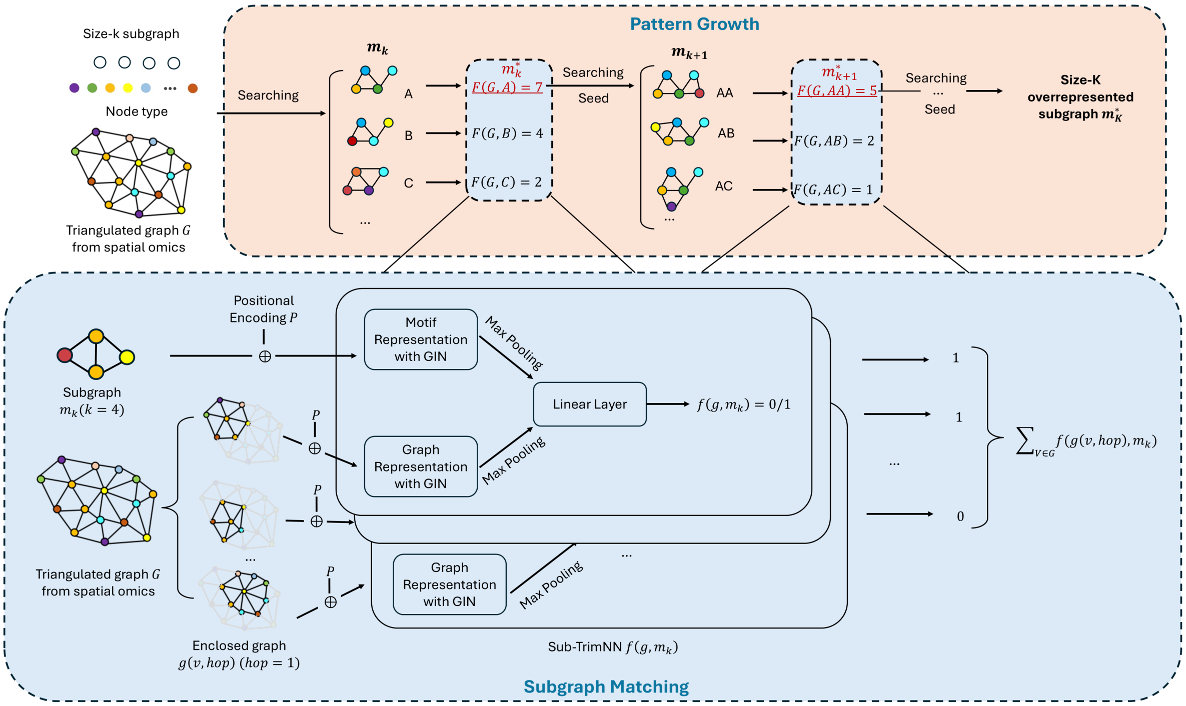

Supplementary Fig. 2 The schema of TrimNN targets challenges of Pattern Growth (top) and Subgraph Matching (bottom). A size-4 subgraph m_k is taken as an example. In the challenge of Pattern Growth, all possible size-4 subgraphs are enumerated to check their occurrence in a triangulated graph G with trained TrimNN function $F(G, m_k)$ from Subgraph Matching. TrimNN summarizes many sub-TrimNN functions $f(g, m_k)$. For each sub-TrimNN, binary prediction on presence (1) or absence (0) is made by m_k on all possible enclosed subgraph g in G. The model uses GIN to learn the representations, plus a positional encoding of the query motif and the target graph, then learns the relations between them.

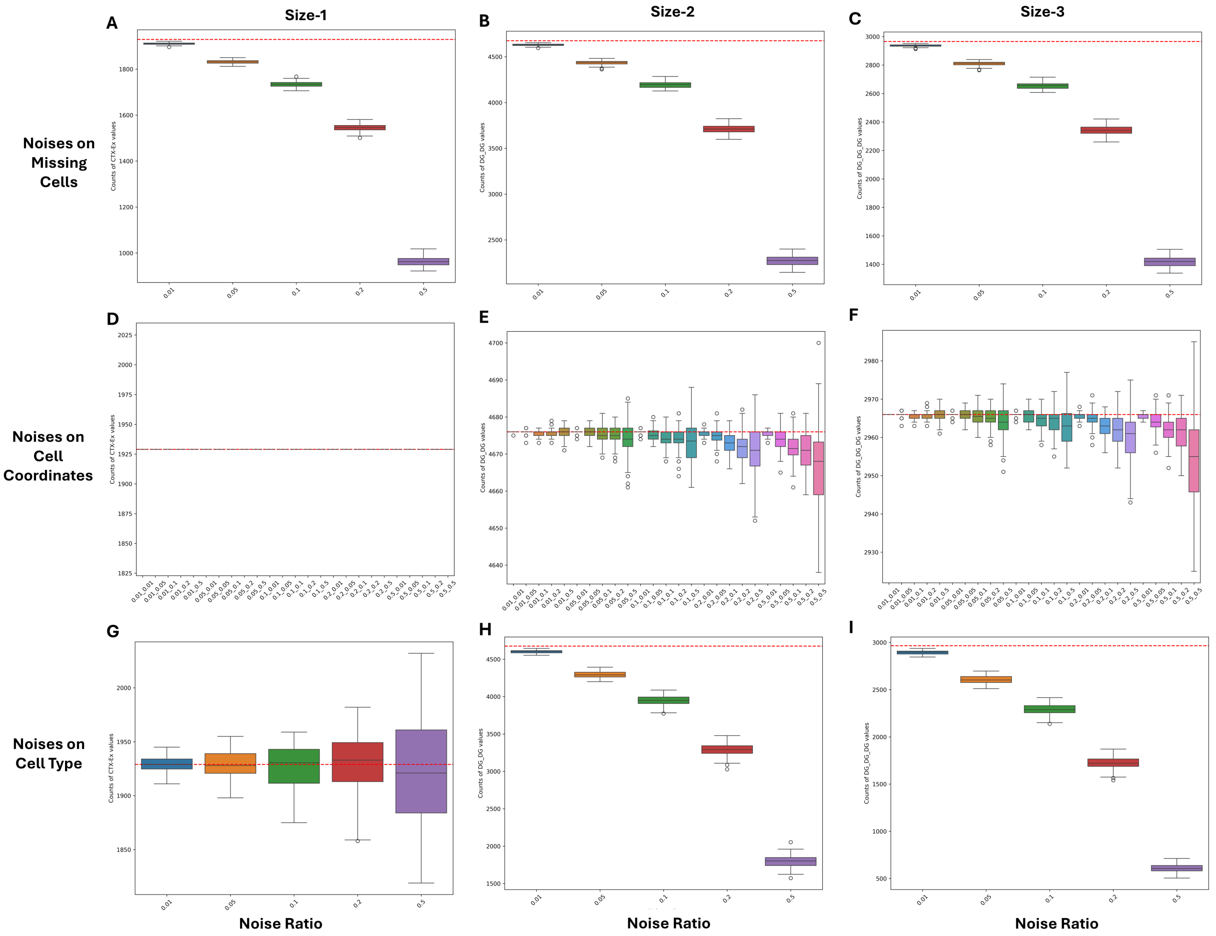

Supplementary Fig. 3 A-C. Simulations of cell missing effects on CC motifs at cell proportions of 0.01, 0.05, 0.1, 0.2, and 0.5, represented as absolute occurrence count of the most frequently occurring CC motif before and after simulated noises within **A.** size-1, **B**. size-2, and **C.** size-3. The red line indicates the absolute occurrence count of the most frequently occurring CC motif in the original data. **D-F**. Simulations of cell coordinate shifting effects on CC motifs at cell proportions of 0.01, 0.05, 0.1, 0.2, and 0.5 with different levels of noises of 0.01, 0.05, 0.1, 0.2, and 0.5. They are represented as the absolute occurrence count of the most frequently occurring CC motif before and after simulated noises within **D.** size-1, **E**. size-2, and **F.** size-3. **G-I**. Simulations of cell type misclassification effects on CC motifs at cell proportions of 0.01, 0.05, 0.1, 0.2, and 0.5, represented as absolute occurrence count of the most frequently occurring CC motif before and after simulated noises within **G.** size-1, **H**. size-2, and **I.** size-3. n=100 samples. On each box, the central mark indicates the median, and the bottom and top edges of the box indicate the 25th and 75th percentiles. The whiskers extend to the most extreme data points without outliers, and the outliers are plotted individually as circles. Source data are provided as a Source Data file.

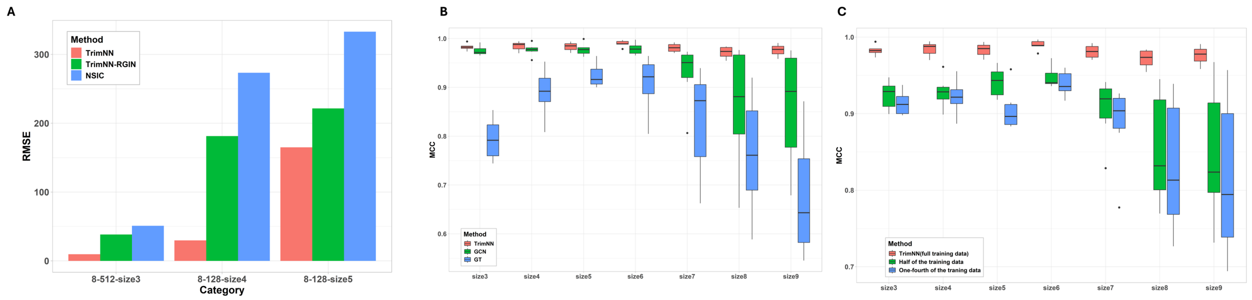

Supplementary Fig. 4 A. Comparison of the performance of TrimNN, TrimNN-RGIN, and NSIC in the abundance of CC motifs with root mean square error (RMSE). **B.** Comparison of the model performances by replacing Graph Isomorphism Network (GIN) in TrimNN with Graph Convolutional Networks (GCN) and Graph Transformer (GT). The X-axis represents different sizes of CC motifs, the Y-axis indicates Matthews Correlation Coefficient (MCC) values (n=3000 samples). **C.** Comparison of the performance of TrimNN trained with full, half, and one-quarter of the training data (n=3000 samples). On each box, the central mark indicates the median, and the bottom and top edges of the box indicate the 25th and 75th percentiles. The whiskers extend to the most extreme data points without outliers, and the outliers are plotted individually as circles. Source data are provided as a Source Data file.

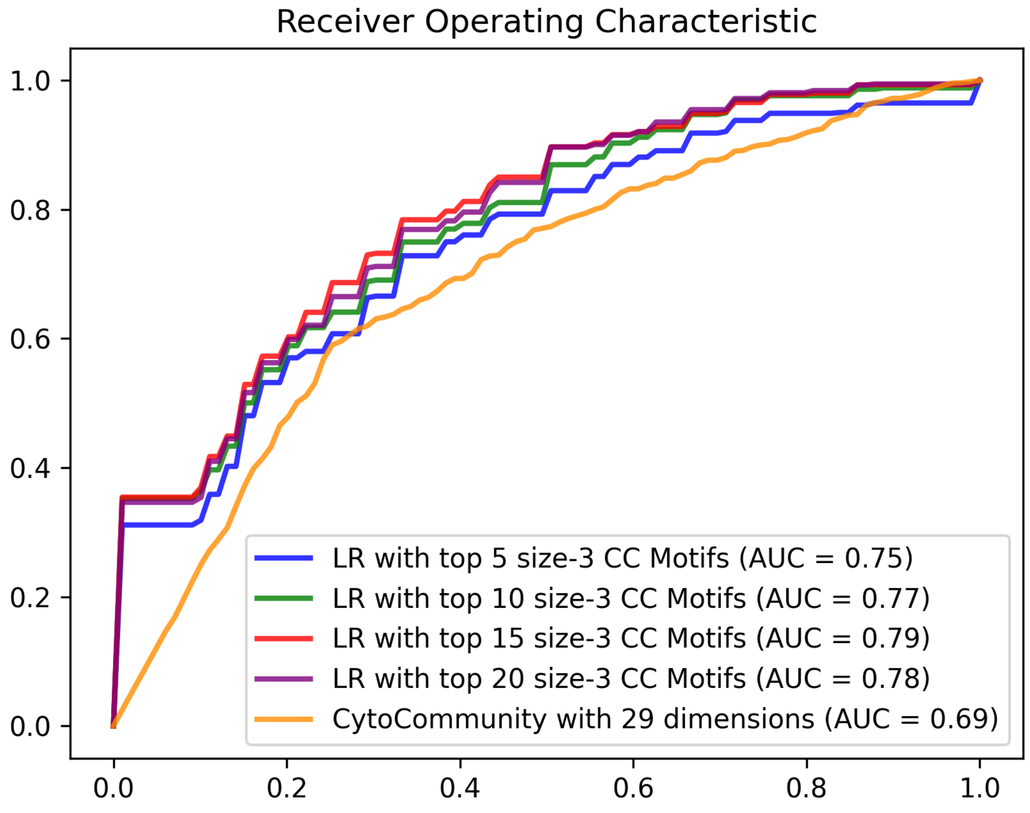

Supplementary Fig. 5 The ROC curves of the Logistics Regression model classify CLR and DII patients using top 5,10,15,20 size-3 CC motifs as features, and CytoCommunity uses 29 dimensions. The Logistic Regression model uses features as motif counts from TrimNN and scales between 0 and 1.

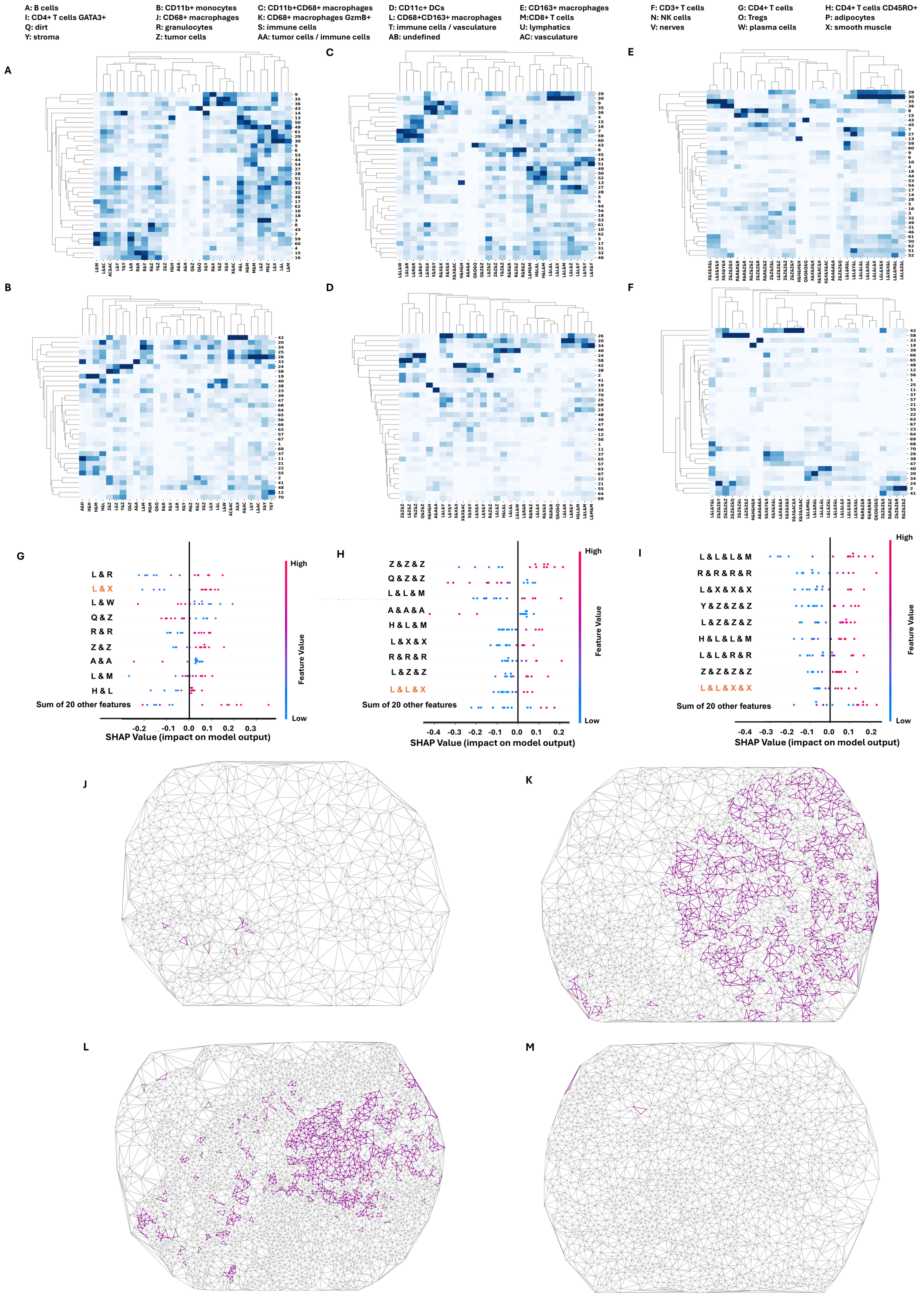
A: B cells
I: CD4+ T cells GATA3+
Q: dirt
Y: stroma
B: CD11b+ monocytes
J: CD68+ macrophages
R: granulocytes
Z: tumor cells
C: CD11b+CD68+ macrophages
K: CD68+ macrophages GzmB+
S: immune cells
AA: tumor cells / immune cells
D: CD11c+ DCs
L: CD68+CD163+ macrophages
T: immune cells / vasculature
AB: undefined
E: CD163+ macrophages
M:CD8+ T cells
U: lymphatics
AC: vasculature
F: CD3+ T cells
N: NK cells
V: nerves
G: CD4+ T cells
O: Tregs
W: plasma cells
H: CD4+ T cells CD45RO+
P: adipocytes
X: smooth muscle
A
B
C
D
E
F
G
H
I
J
K
L
M
SHAP Value (impact on model output)
Feature Value
High
Low
Sum of 20 other features

Supplementary Fig. 6 The clustering map of the abundance of **A**. size-2, **C**. size-3, and **E**. size-4 CC motifs among DII patient samples and **B**. size-2, **D**. size-3, and **F**. size-4 CC motifs among CLR patient samples. The SHAP value of the Logistics Regression model using top 29 **G**. size-2, **H**. size-3, and **I**. size-4 CC motifs as features. The locations of the 'tumor & immune & immune' motif (marked as purple, others grey) are visualized in **J**. patient 35 (CLR) on spot 70 B and **K**. patient 9 (DII) on spot 18 B. The locations of the 'B cells & B cells & B cells' motif (marked as purple, others grey) are visualized in **L**. patient 28 (CLR) on spot 55 A and **M**. patient 14 (DII) on spot 28. Source data are provided as a Source Data file.

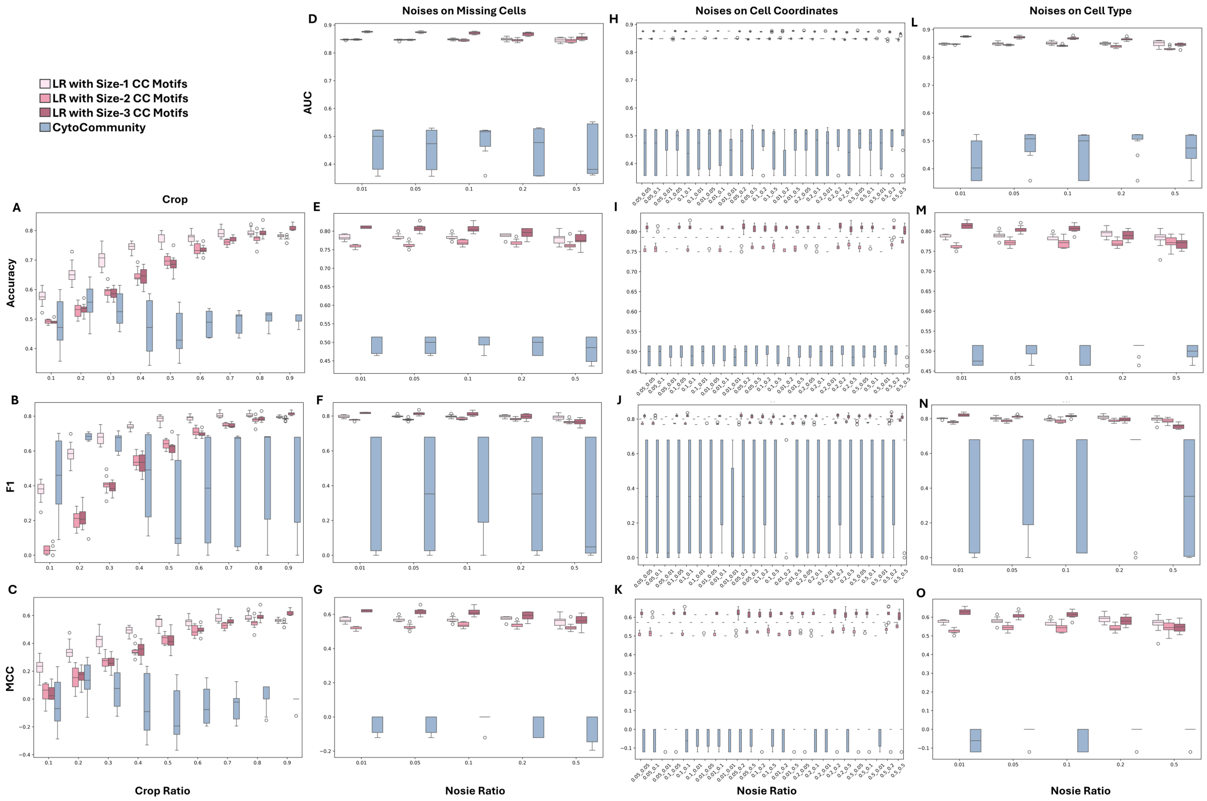

Supplementary Fig. 7 Generalizability of the trained model testing on random cropping of the samples. The X-axis is the ratio of width and height of the original ROI, and the Y-axis is **A.** accuracy, **B.** F1 score, and **C**. MCC. AUC is shown in Figure 3C in the main text. Generalizability of the trained model testing on distorted samples with noises from missing cells. The X-axis is the noise ratio, and the Y-axis is **D.** AUC, **E.** accuracy, **F.** F1 score, and **G**. MCC. Generalizability of the trained model testing on distorted samples with noises from cell coordinates shifting. The X-axis is the noise ratio, and the Y-axis is **H.** AUC, **I.** accuracy, **J.** F1 score, and **K**. MCC. Generalizability of the trained model testing on distorted samples with noises from cell type misclassification. The X-axis is the noise ratio, and the Y-axis is **L.** AUC, **M.** accuracy, **N.** F1 score, and **O**. MCC. n=100 samples. On each box, the central mark indicates the median, and the bottom and top edges of the box indicate the 25th and 75th percentiles. The whiskers extend to the most extreme data points without outliers, and the outliers are plotted individually as circles. Source data are provided as a Source Data file.

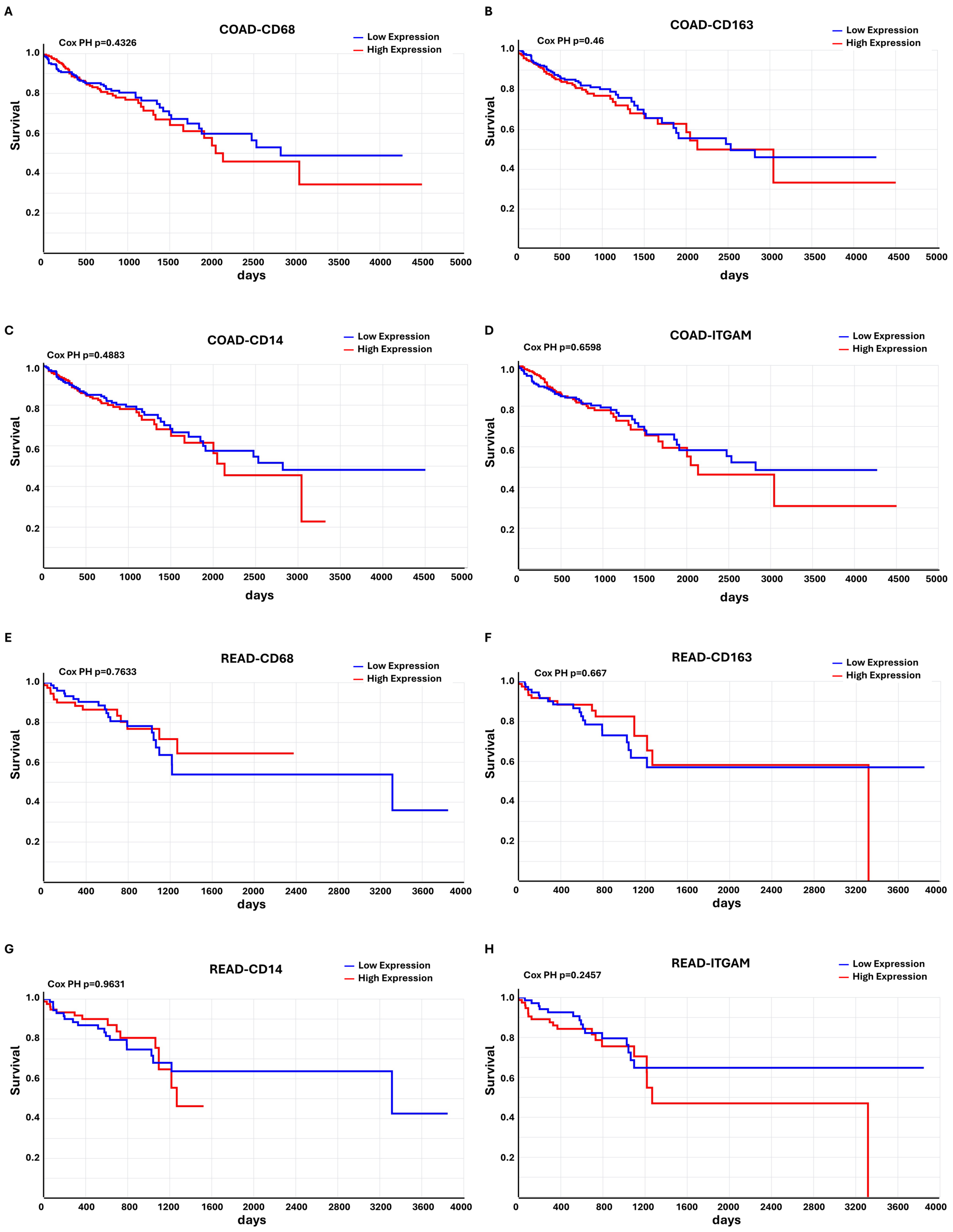
A
COAD-CD68
Cox PH p=0.4326
Low Expression
High Expression
Survival
days
B
COAD-CD163
Cox PH p=0.46
Low Expression
High Expression
Survival
days
C
COAD-CD14
Cox PH p=0.4883
Low Expression
High Expression
Survival
days
D
COAD-ITGAM
Cox PH p=0.6598
Low Expression
High Expression
Survival
days
E
READ-CD68
Cox PH p=0.7633
Low Expression
High Expression
Survival
days
F
READ-CD163
Cox PH p=0.667
Low Expression
High Expression
Survival
days
G
READ-CD14
Cox PH p=0.9631
Low Expression
High Expression
Survival
days
H
READ-ITGAM
Cox PH p=0.2457
Low Expression
High Expression
Survival
days

Supplementary Fig. 8 Survival curves associated with CD68^{+}CD163^{+} Macrophage in TCGA. Survival Curves of high vs. low expression of CD68^{+}CD163^{+} Macrophage marker genes: **A.** CD68, **B.** CD163, **C.** CD14, **D.** ITGAM in TCGA-COAD. Survival Curves of high vs. low expression of CD68^{+}CD163^{+} Macrophage marker genes: **E.** CD68, **F.** CD163, **G.** CD14, **H.** ITGAM in TCGA-READ. The p-values were derived from the two-sided Wald test.

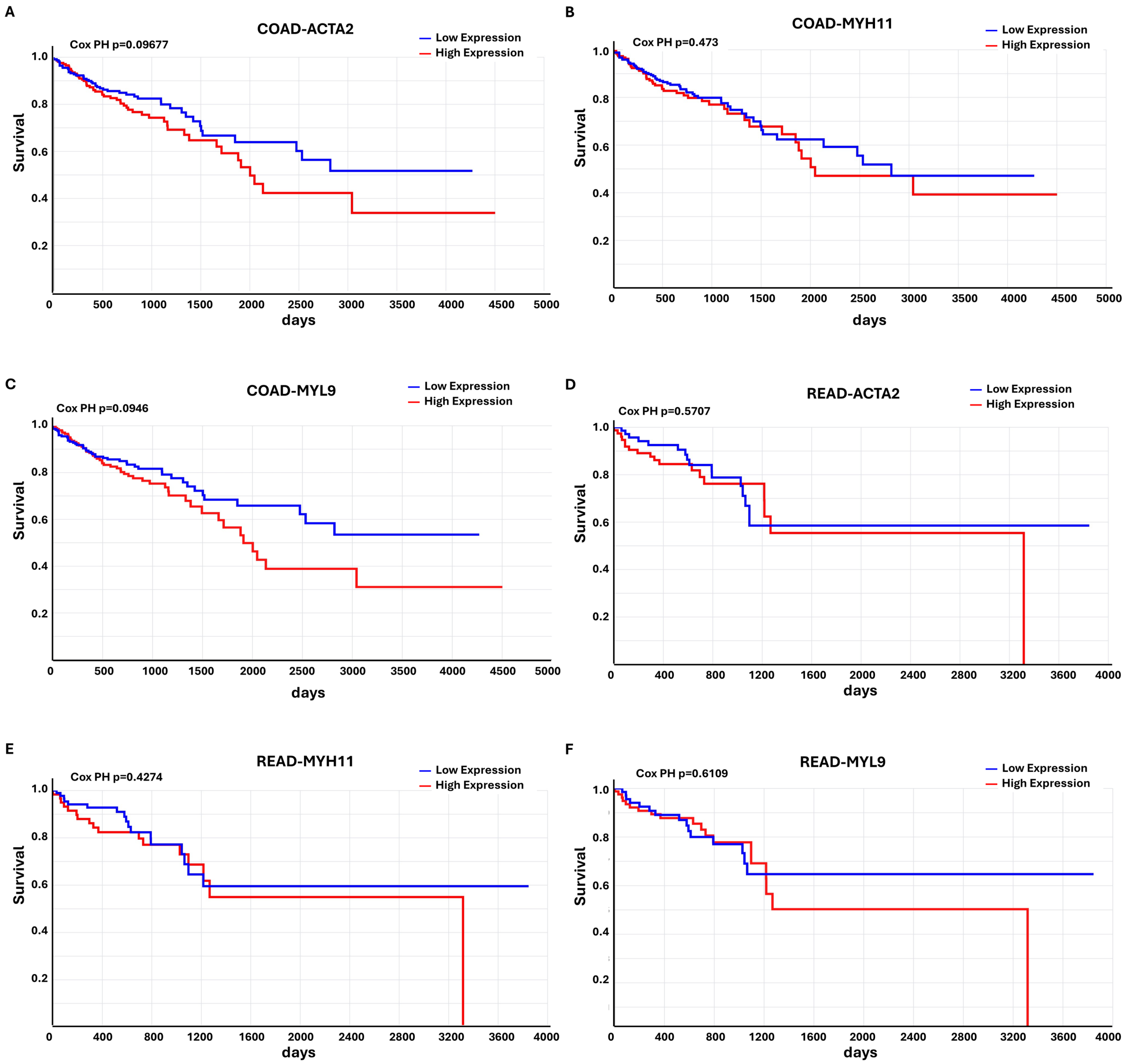

Supplementary Fig. 9 Survival curves associated with smooth muscle in TCGA. Survival curves of high vs. low expression of smooth muscle marker genes: **A**. ACTA2, **B**. MYH11, and **C**. MYL9 in TCGA-COAD. Survival curves of high vs. low expression of smooth muscle marker genes: **D**. ACTA2, **E**. MYH11, and **F**. MYL9 in TCGA-READ. The p-values were derived from the two-sided Wald test.

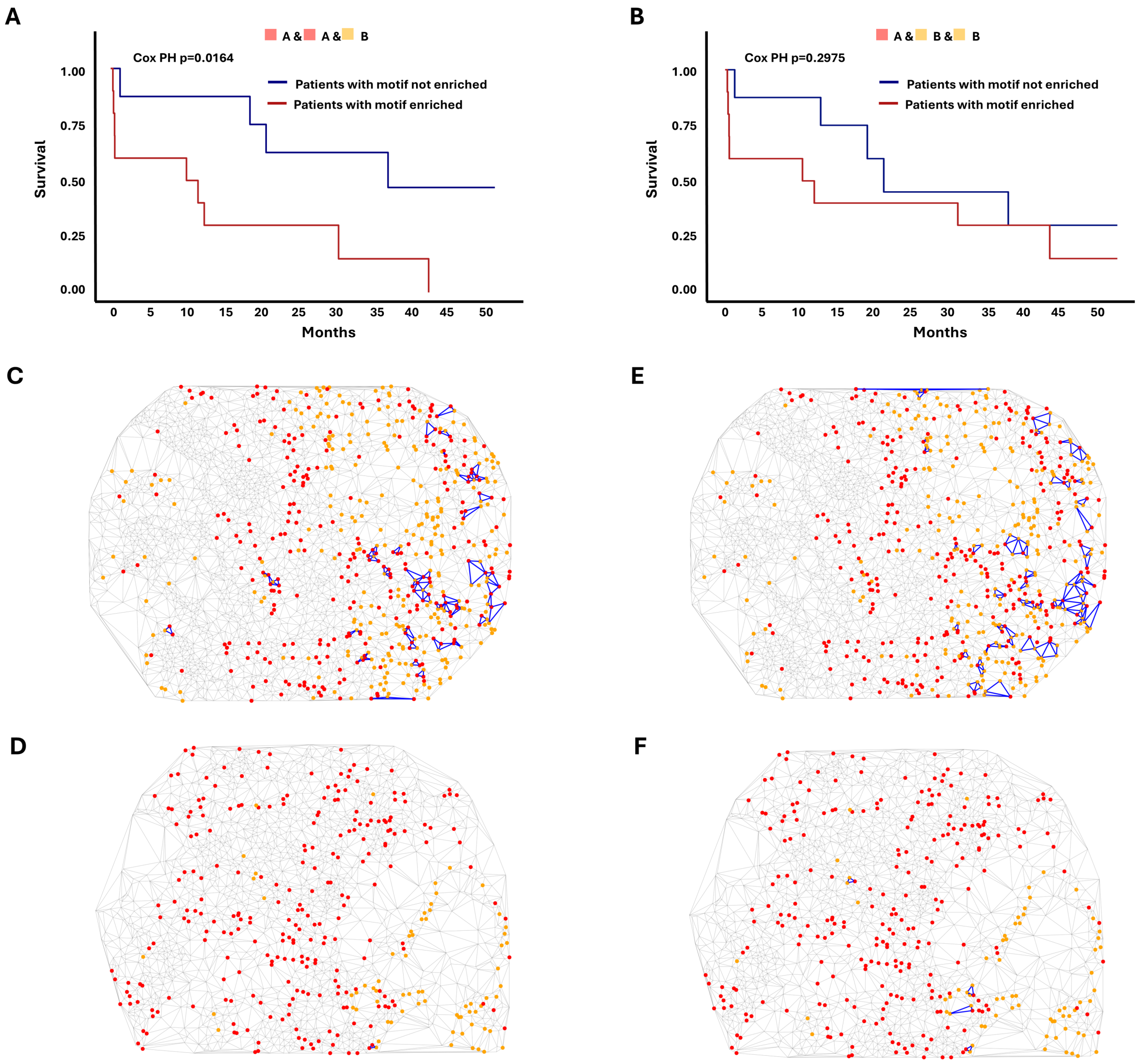

Supplementary Fig. 10 Survival curves of DII patients with and without enriched CC motifs, including size-3 **A**. 'A & A & B' and **B**. 'A & B & B'. The p-values were derived from the two-sided Wald test. Here cell type CD68+CD163+ macrophages are denoted as cell type 'A', and cell type smooth muscle is denoted as cell type 'B'. The visualization of spatial locations of the size-3 motif 'A & A & B' in **C.** DII spot 5A and **D.** CLR spot 16A. The visualization of spatial locations of the size-3 motif 'A & B & B' in **E.** DII spot 5A and **F.** CLR spot 16A.,

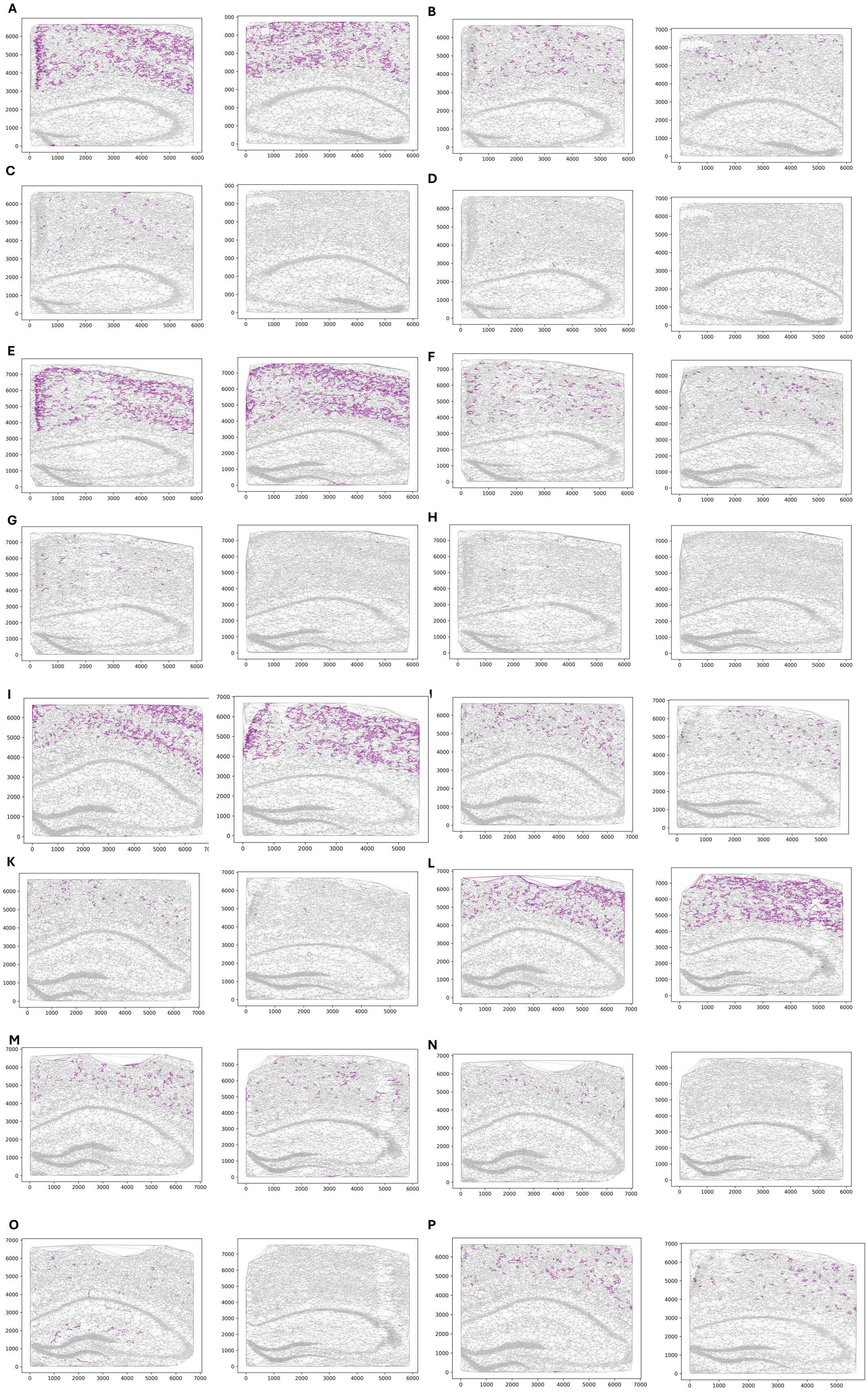
A
B
C
D
E
F
G
H
I
J
K
L
M
N
O
P

Supplementary Fig.11 Representative CC motifs in triangulation graph in disease (left column) and control (right column) samples, including localization of motifs **A.** ‘CCC’, **B.** ‘CCM’**, C.** ‘CMM’, and **D.** ‘MMM’ in eight-month-old sample replicate 1, motifs **E.** ‘CCC’, **F.** ‘CCM’**, G.** ‘CMM’, and **H.** ‘MMM’ in eight-month-old sample replicate 2, motifs **I.** ‘CCC’**, J.** ‘CCM’, and **K.** ‘CMM’ in thirteen-month-old sample replicate 1, motifs **L.** ‘CCC’, **M.** ‘CCM’**, N.** ‘CMM’, and **O.** ‘MMM’ in thirteen-month-old sample replicate 2, Size-4 CC motif **P**. ‘CCCM’ in thirteen-month-old sample replicate 1.

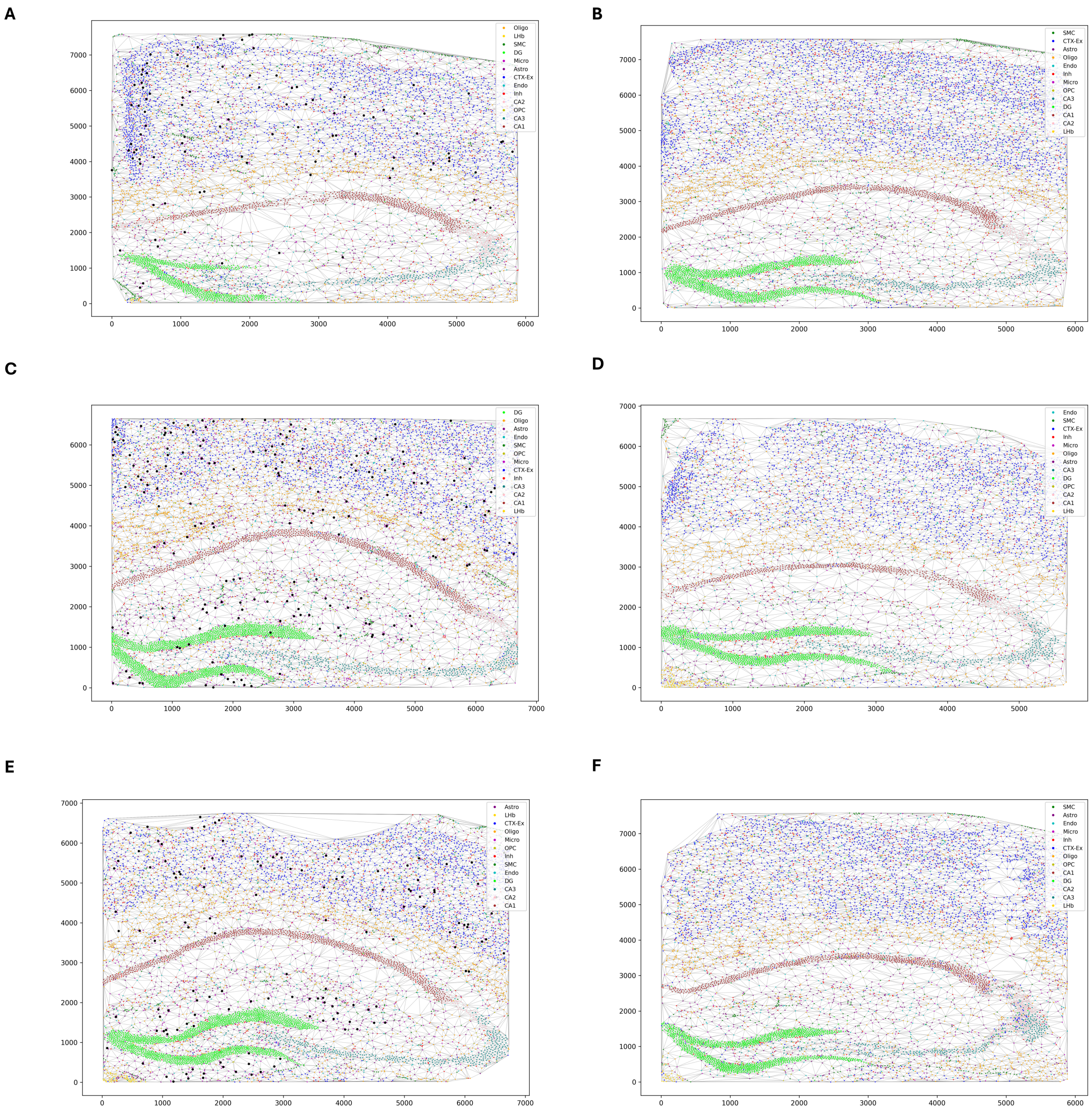

Supplementary Fig. 12 The triangulated graphs as cellular communities of all other samples other than thirteen-month-old samples in the main figure, including eight-month-old replicate 1 in **A.** disease and **B.** control samples; eight-month-old replicate 2 **C.** disease and **D.** control samples; thirteen-month-old replicate 2 **E.** disease and **F.** control samples.

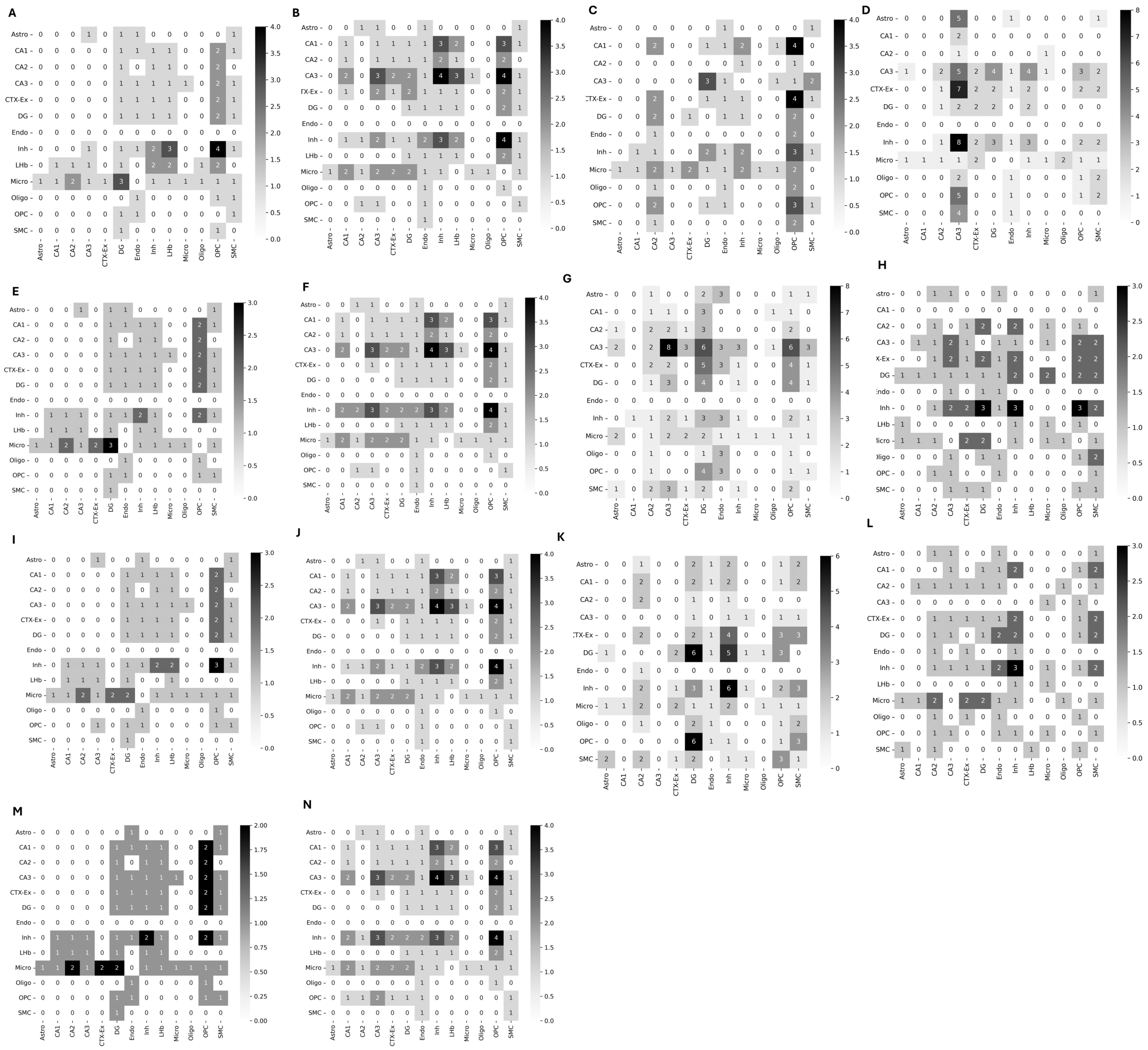

Supplementary Fig. 13 CellChat results as a matrix between different cell types in CC motif regions (motif and its 3-hop) and non-motif regions. Number of links in non-motif 'CCC' regions in **A.** eight-month-old samples and **B.** thirteen-month-old samples. Number of links in motif 'CCM' regions in **C.** eight-month-old samples and **D.** thirteen-month-old samples. Number of links in non-motif 'CCM' regions in **E.** eight-month-old samples and **F.** thirteen-month-old samples. The number of links in motif 'CMM' regions in **G.** eight-month-old samples and **H.** thirteen-month-old samples. The number of links in non-motif 'CMM' regions in **I.** eight-month-old samples and **J.** thirteen-month-old samples. The number of links in motif 'MMM' regions in **K.** eight-month-old samples and **L.** thirteen-month-old samples. The number of links in non-motif 'MMM' regions in **M.** eight-month-old samples and **N.** thirteen-month-old samples. The number of links is defined in CellChat software by cellchat@net$count. Source data are provided as a Source Data file.

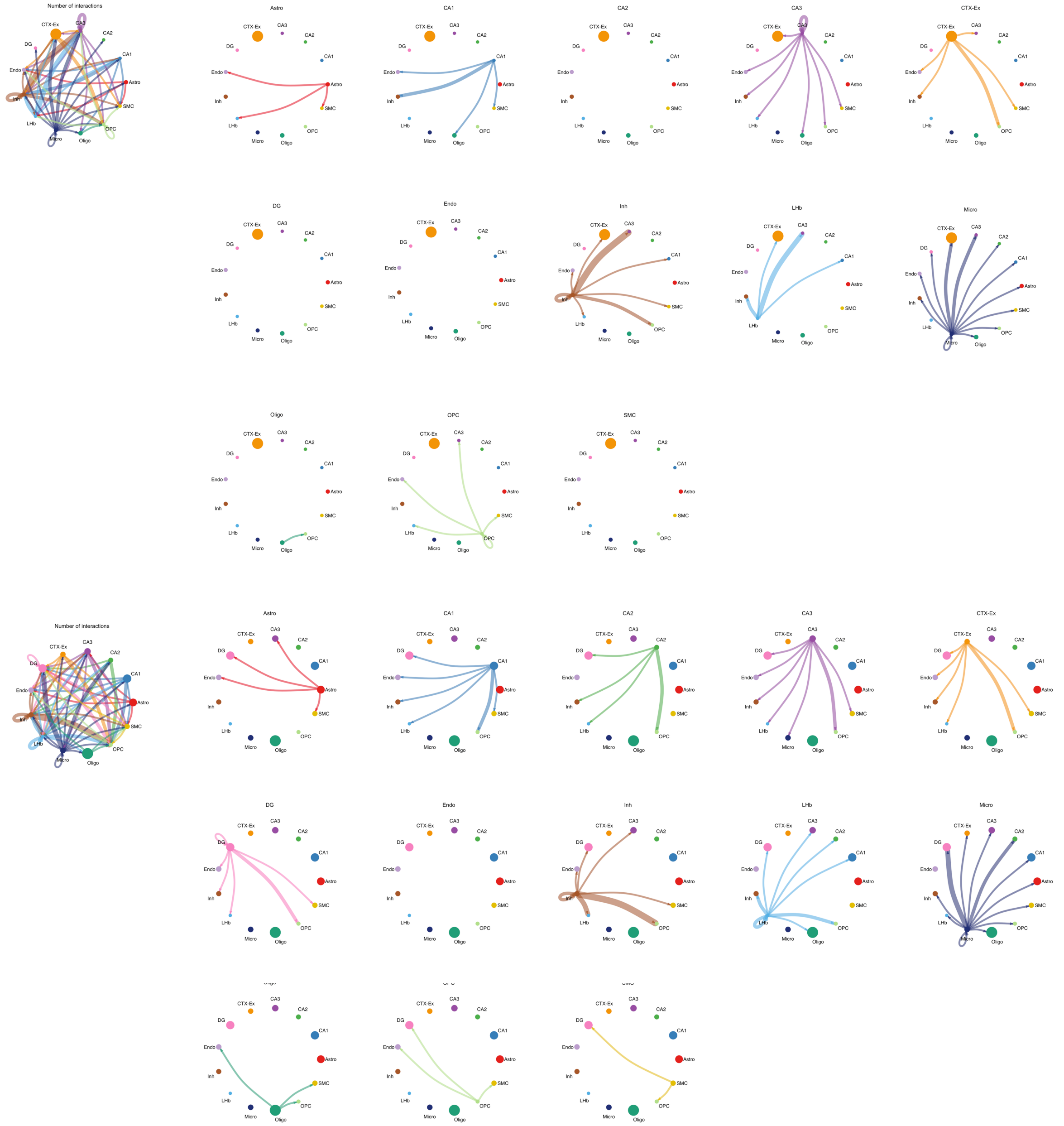

Supplementary Fig. 14 Visualization of CellChat results on each cell type between in ‘CCC’ motif regions (motif and its 3-hop, top) and non-motif regions (bottom) in eight-month-old samples.

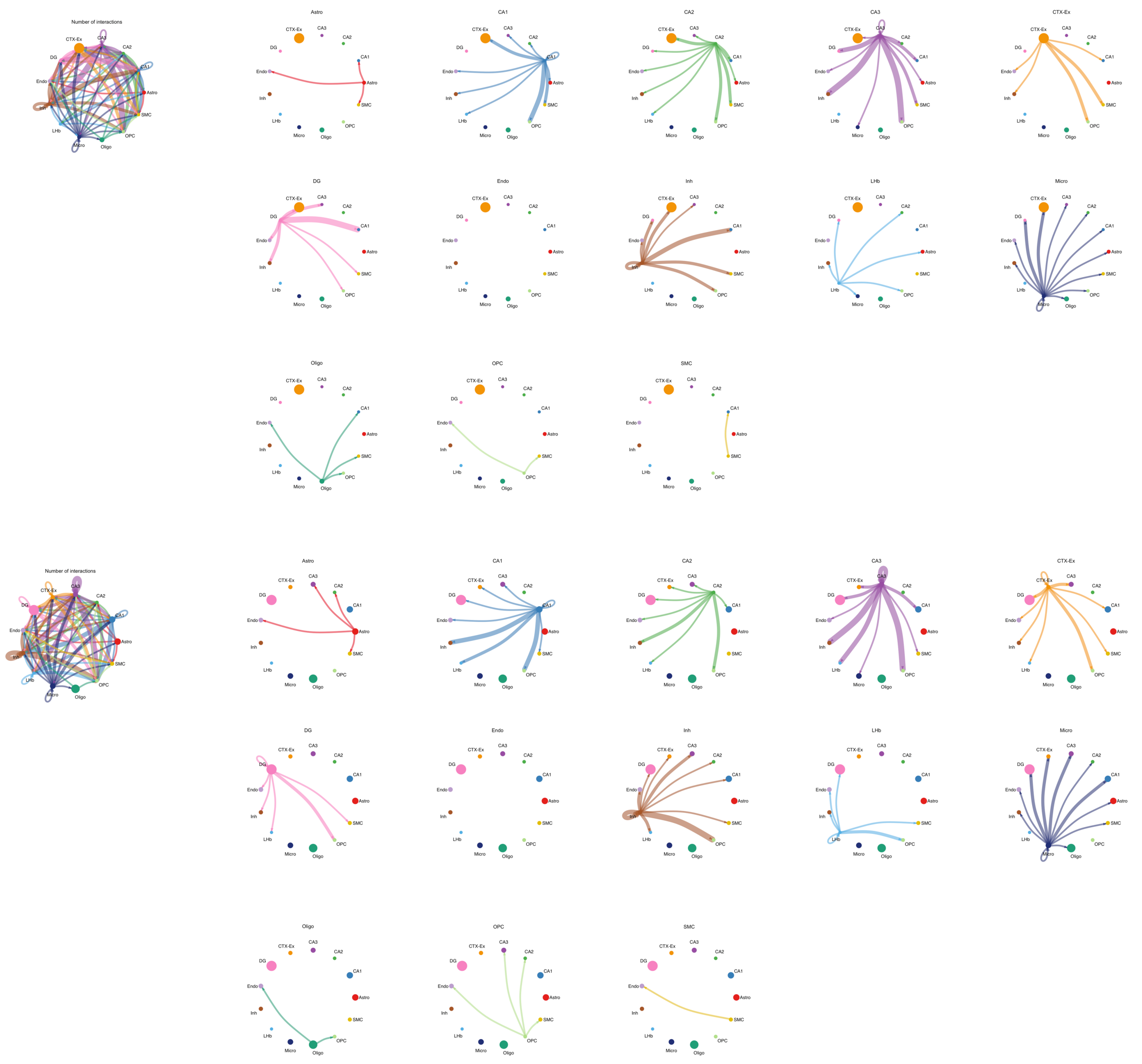

Supplementary Fig. 15 Visualization of CellChat results on each cell type between in 'CCC' motif regions (motif and its 3-hop, top) and non-motif regions (bottom) in thirteen-month-old samples.

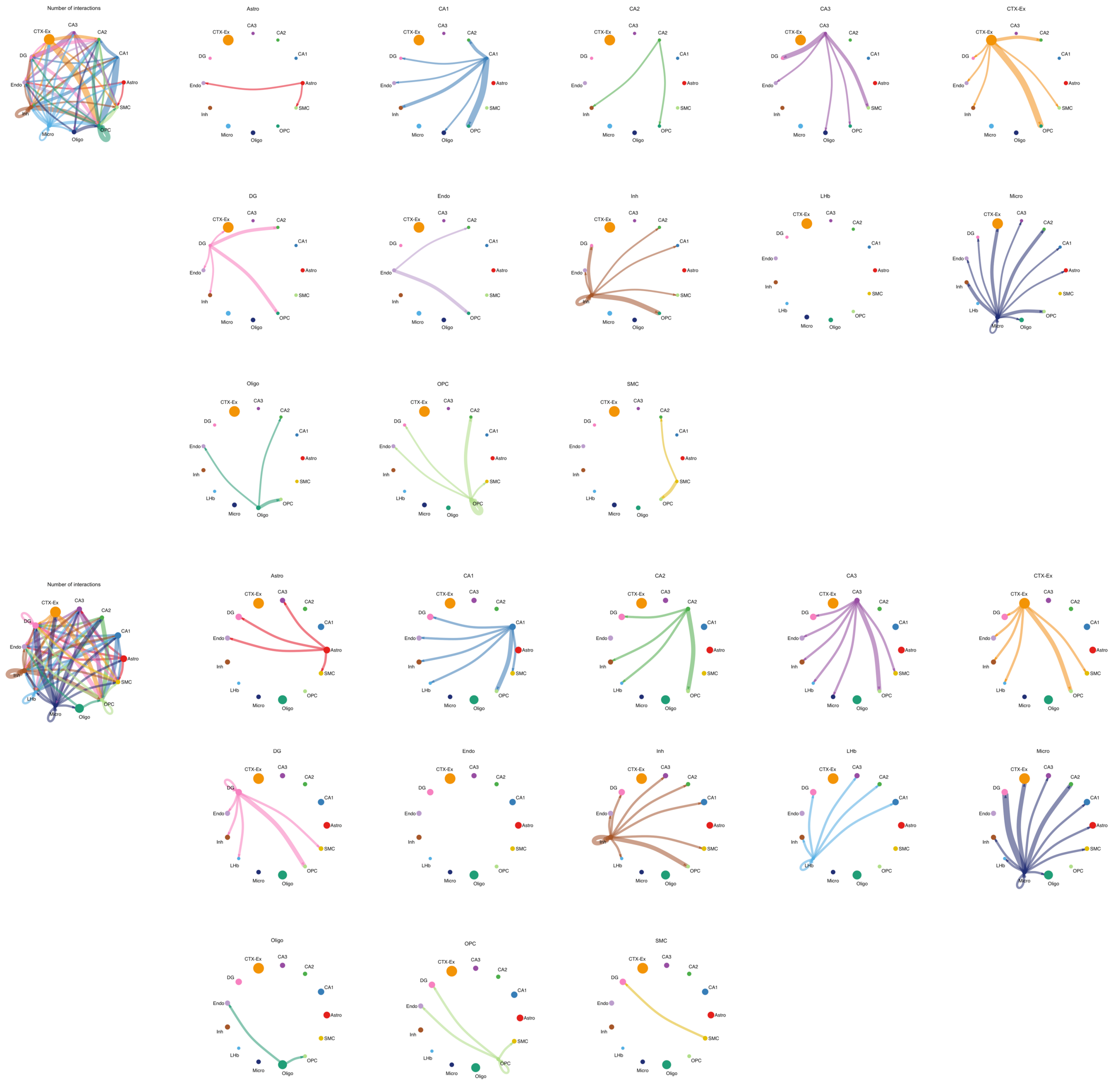

Supplementary Fig. 16 Visualization of CellChat results on each cell type between in ‘CCM’ motif regions (motif and its 3-hop, top) and non-motif regions (bottom) in eight-month-old samples.

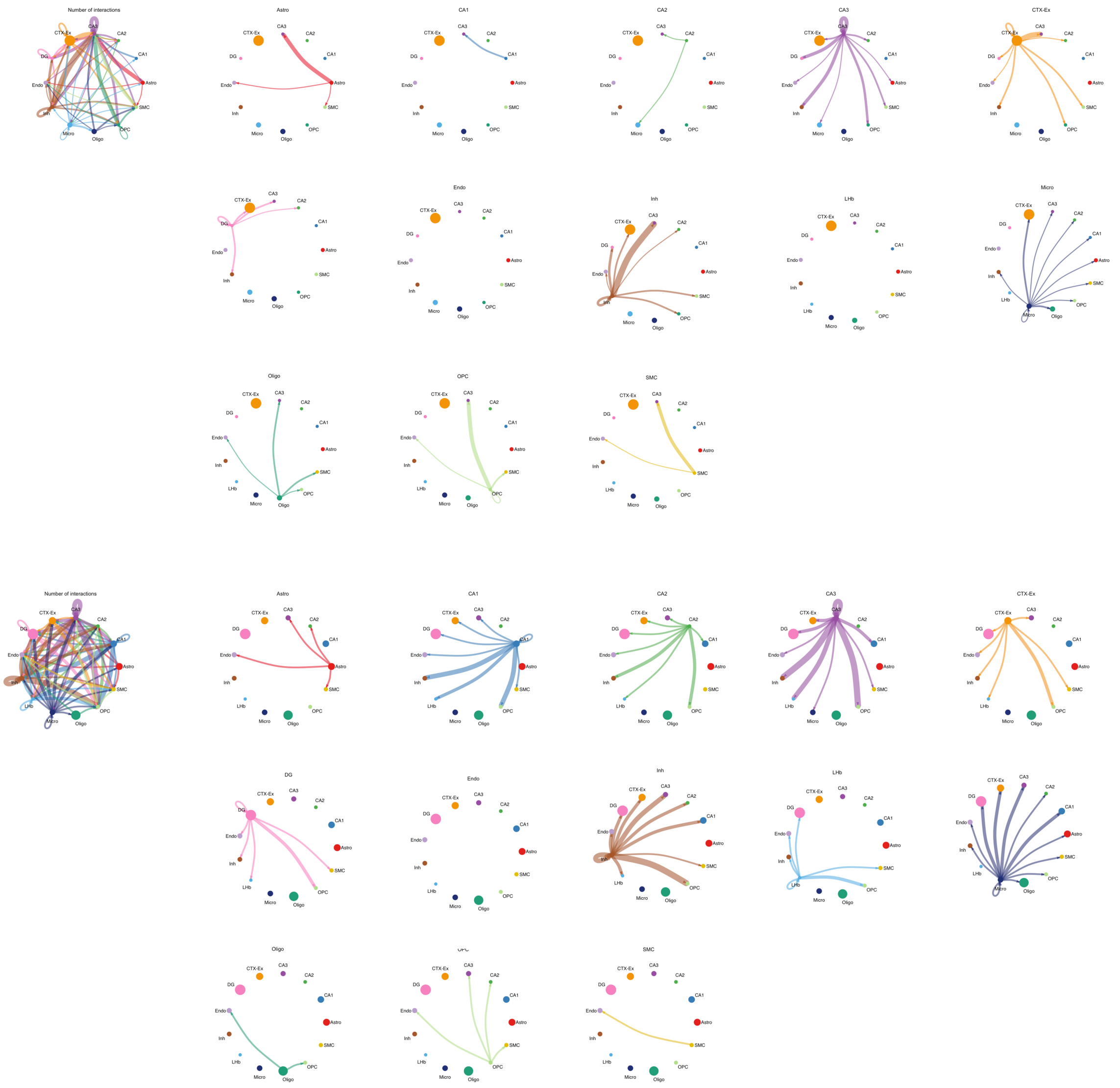

Supplementary Fig. 17 Visualization of CellChat results on each cell type between in 'CCM' motif regions (motif and its 3-hop, top) and non-motif regions (bottom) in thirteen-month-old samples.

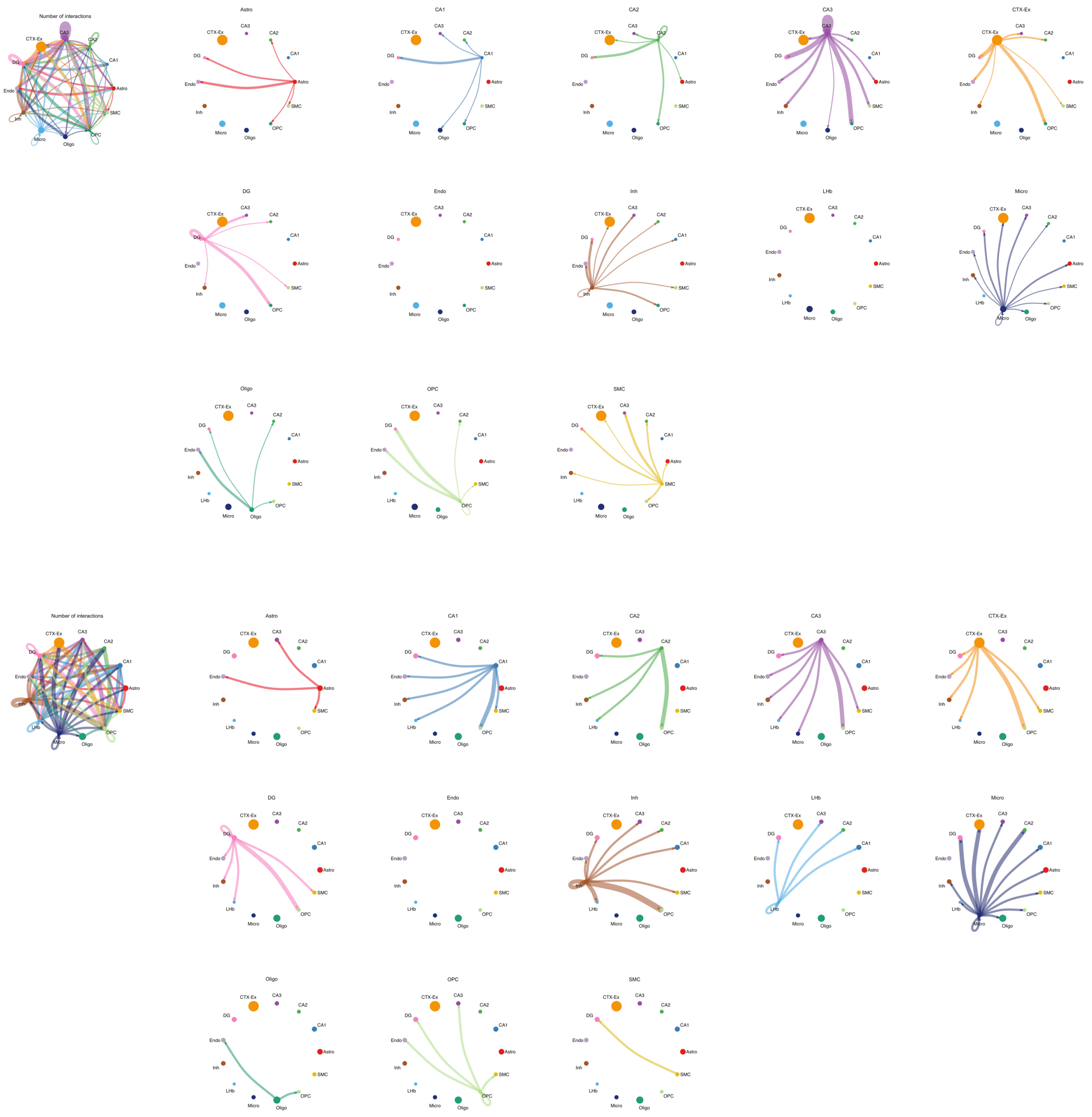

Supplementary Fig. 18 Visualization of CellChat results on each cell type between in 'CMM' motif regions (motif and its 3-hop, top) and non-motif regions (bottom) in eight-month-old samples.

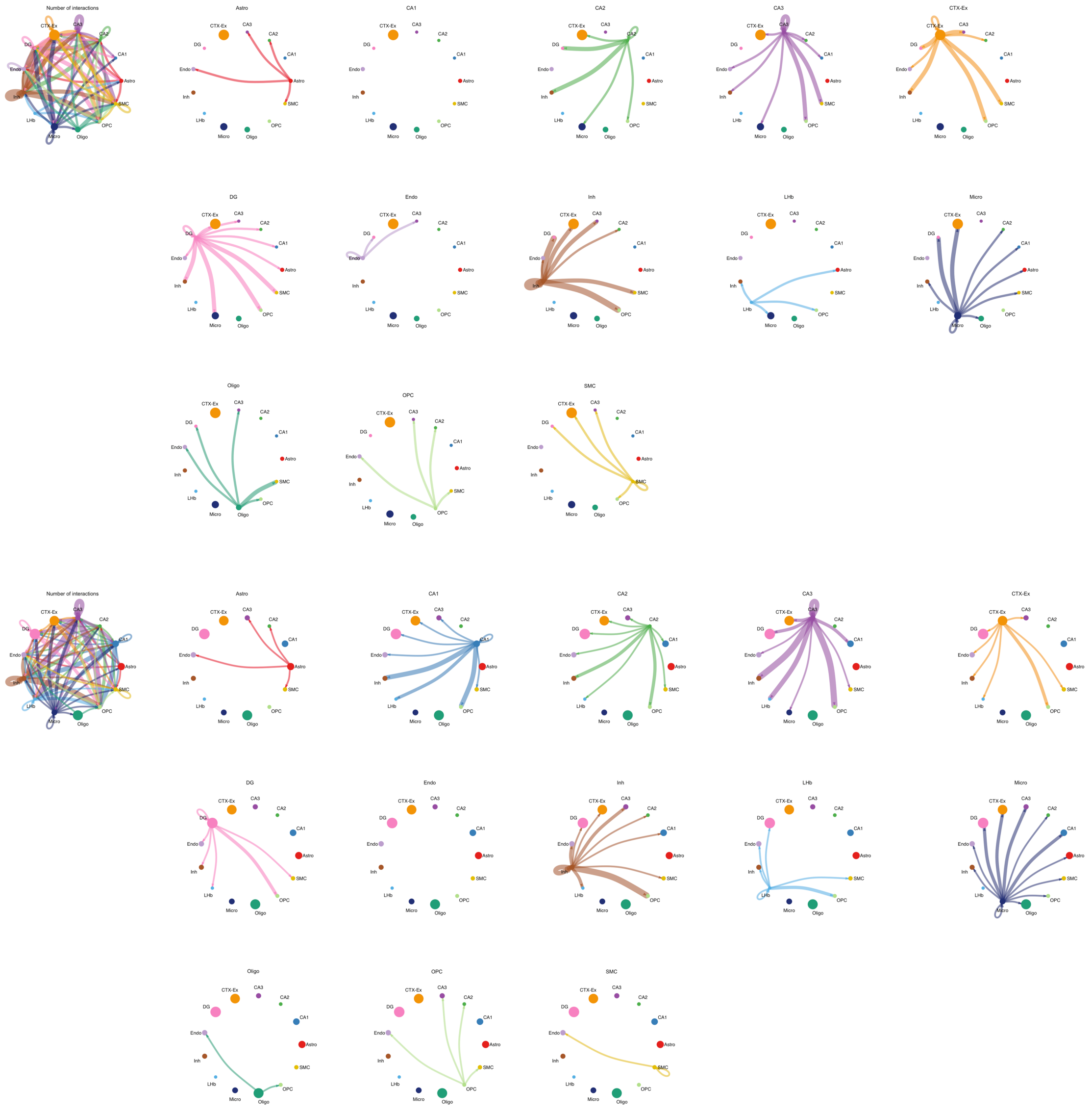

Supplementary Fig. 19 Visualization of CellChat results on each cell type between in ‘CMM’ motif regions (motif and its 3-hop, top) and non-motif regions (bottom) in thirteen-month-old samples.

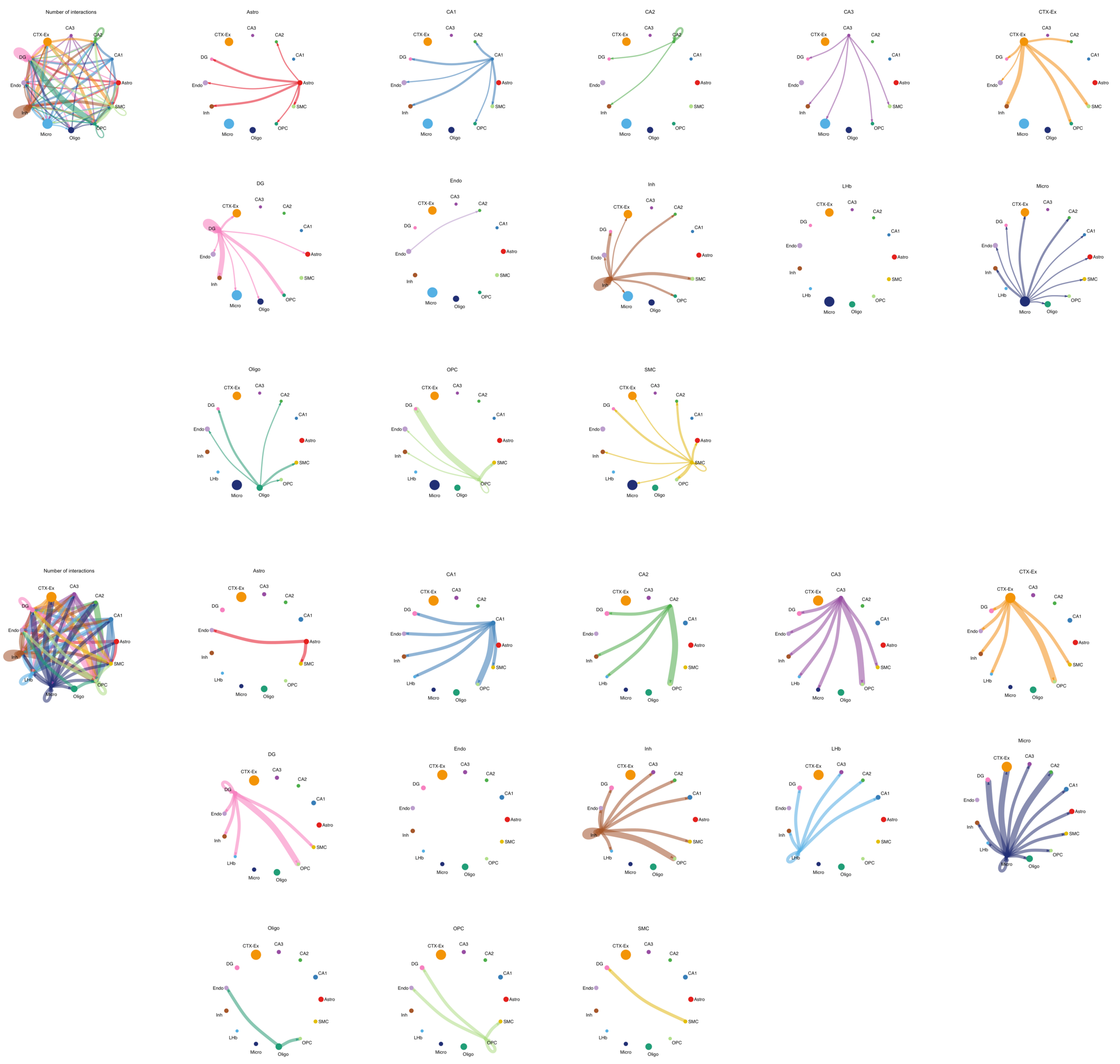

Supplementary Fig. 20 Visualization of CellChat results on each cell type between in ‘MMM’ motif regions (motif and its 3-hop, top) and non-motif regions (bottom) in eight-month-old samples.

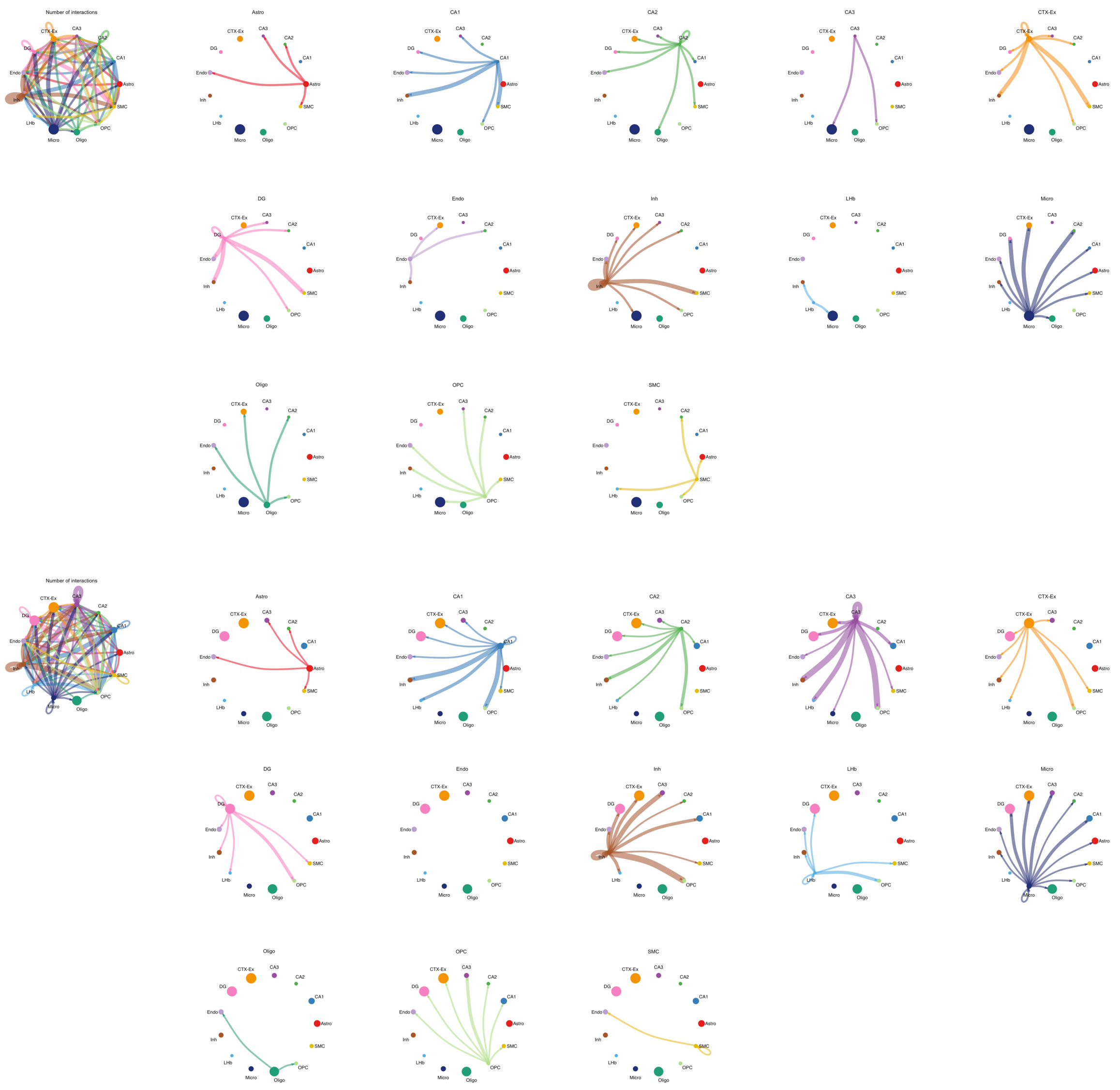

Supplementary Fig. 21 Visualization of CellChat results on each cell type between in ‘MMM’ motif regions (motif and its 3-hop, top) and non-motif regions (bottom) in thirteen-month-old samples.

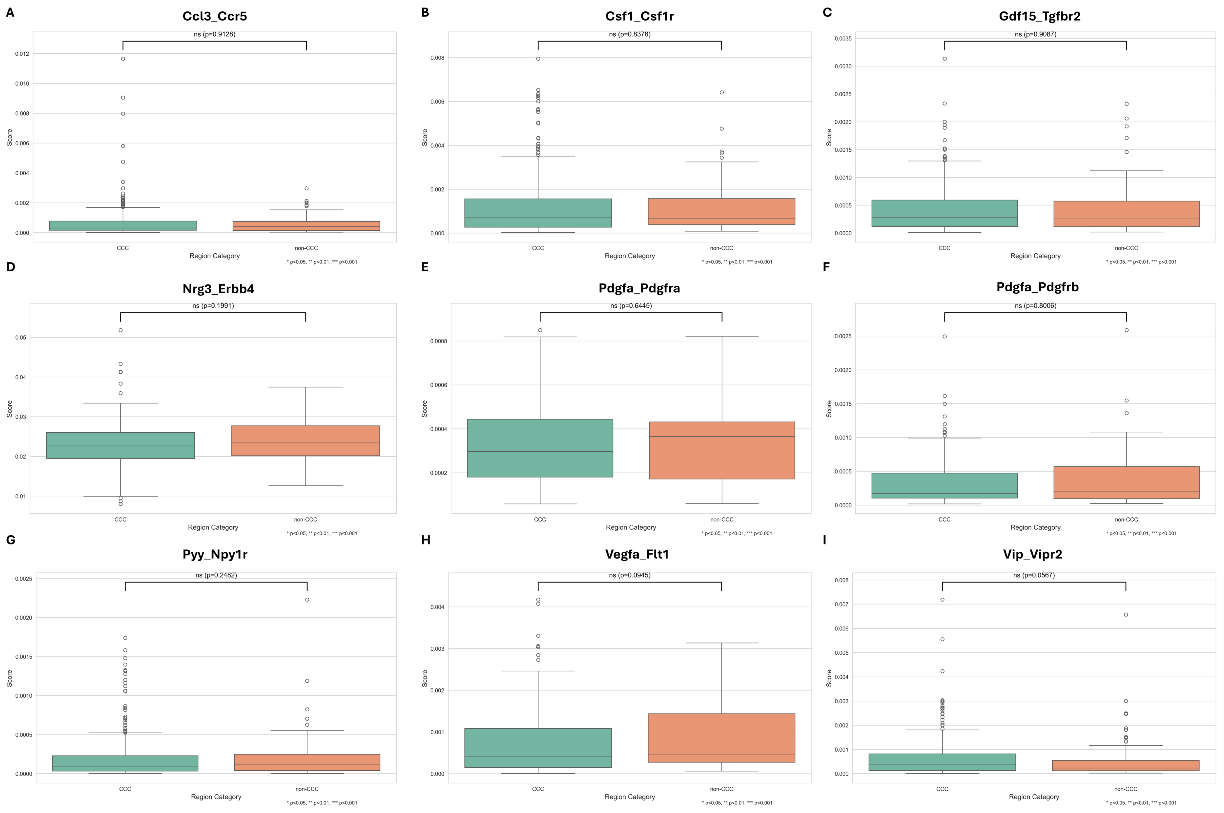

Supplementary Fig. 22 Comparison of Cortex (C)-to-Microglia communication scores between CCC (left) and non-CCC (right) regions mediated by different Ligand-Receptor (L-R) pairs using DeepTalk. The box plot shows the differences in L-R pair **A**. Ccl3-Ccr5, **B**. Csf1-Csf1r, **C**. Gdf15-Tgfbr2, **D**. Nrg3-Erbb4, **E**. Pdgfa-Pdgfra, **F**. Pdgfa-Pdgfrb, **G**. Pyy-Npy1r, **H**. Vegfa-Flt1, and **I**. Vip-Vipr2. The two-sided Mann-Whitney U-test is used to test the significance level. Source data are provided as a Source Data file.

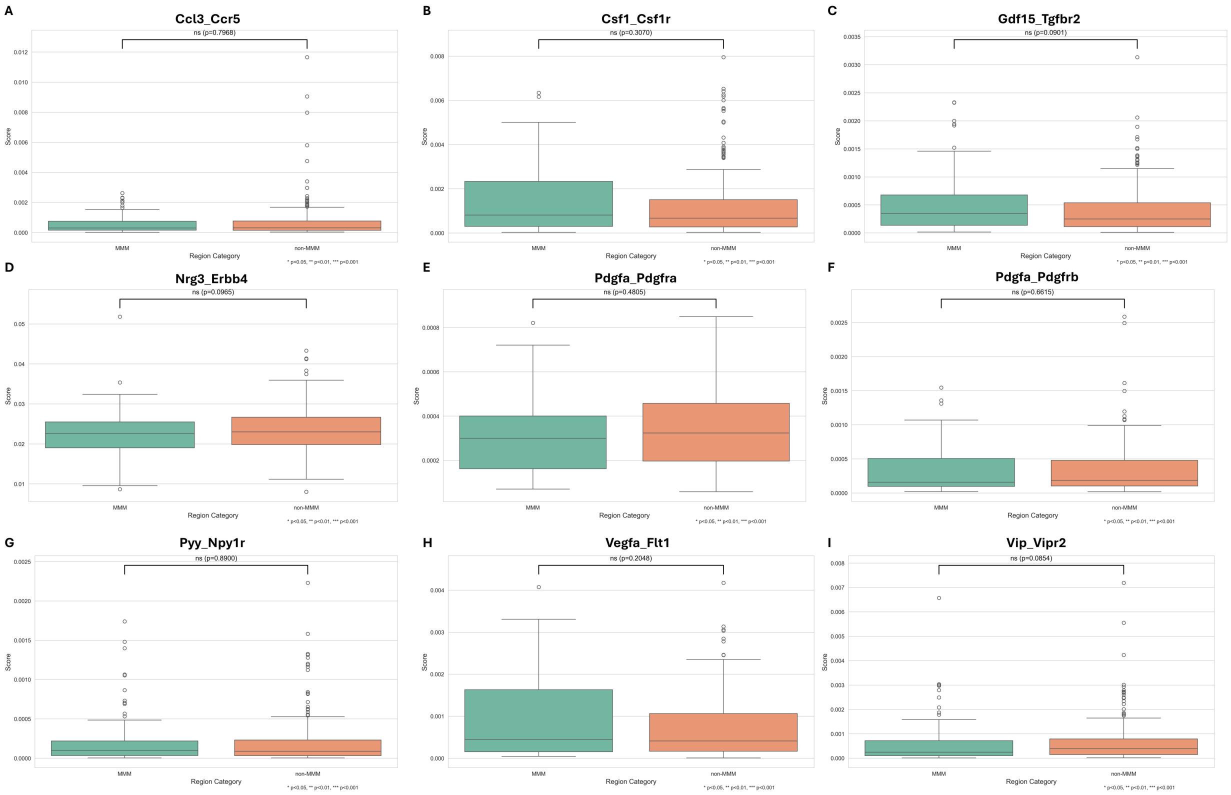

Supplementary Fig. 23 Comparison of Cortex-to-Microglia (M) communication scores between MMM (left) and non-MMM (right) regions mediated by different Ligand-Receptor (L-R) pairs using DeepTalk. The box plot shows the differences in L-R pair **A**. Ccl3-Ccr5, **B**. Csf1-Csf1r, **C**. Gdf15-Tgfbr2, **D**. Nrg3-Erbb4, **E**. Pdgfa-Pdgfra, **F**. Pdgfa-Pdgfrb, **G**. Pyy-Npy1r, **H**. Vegfa-Flt1, and **I**. Vip-Vipr2. The two-sided Mann-Whitney U-test is used to test the significance level. Source data are provided as a Source Data file.

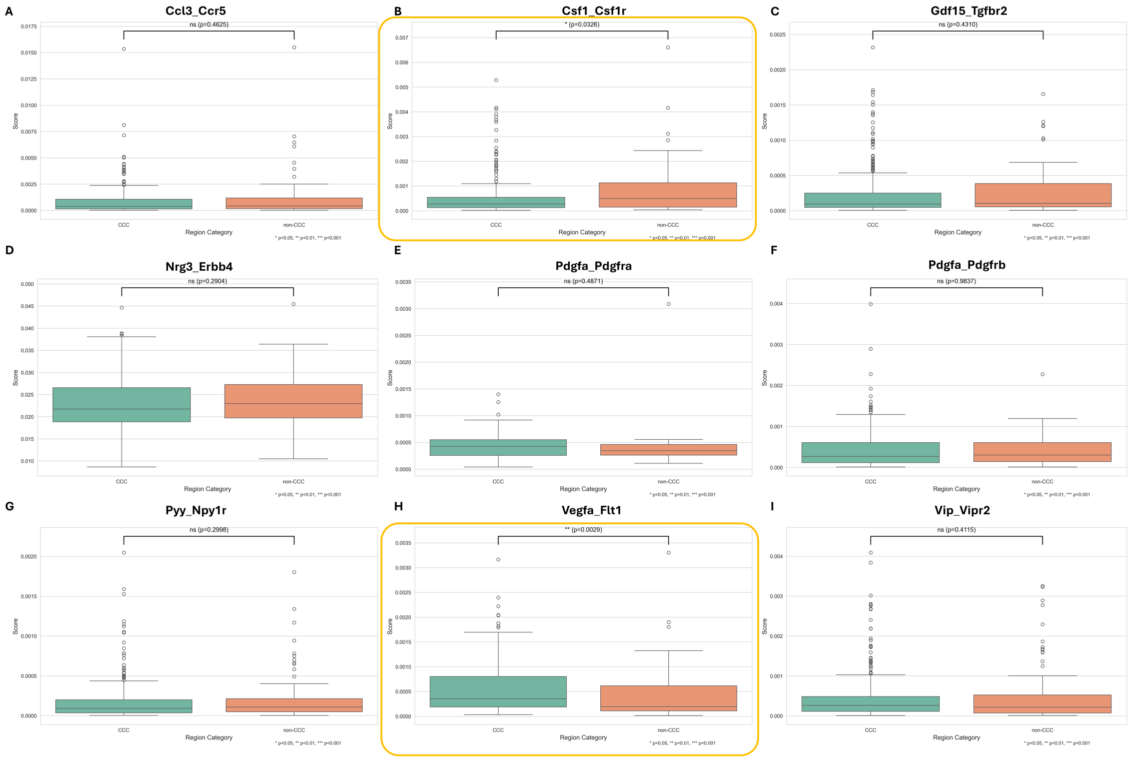

Supplementary Fig. 24 Comparison of Microglia-to- Cortex (C) communication scores between CCC (left) and non-CCC (right) regions mediated by different Ligand-Receptor (L-R) pairs using DeepTalk. The box plot shows the differences in L-R pair **A**. Ccl3-Ccr5, **B**. Csf1-Csf1r, **C**. Gdf15-Tgfbr2, **D**. Nrg3-Erbb4, **E**. Pdgfa-Pdgfra, **F**. Pdgfa-Pdgfrb, **G**. Pyy-Npy1r, **H**. Vegfa-Flt1, and **I**. Vip-Vipr2. The two-sided Mann-Whitney U-test is used to test the significance level. L-R pairs in a yellow rectangle are statistically significant. Source data are provided as a Source Data file.

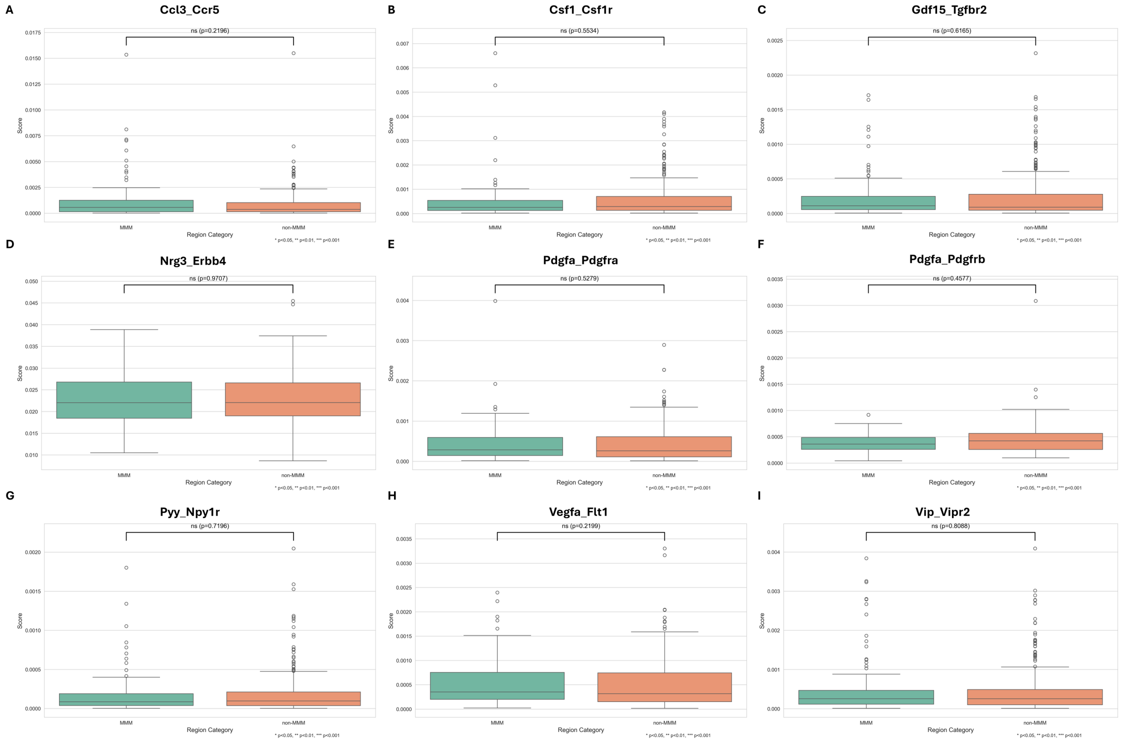

Supplementary Fig. 25 Comparison of Microglia (M)-to- Cortex communication scores between MMM (left) and non-MMM (right) regions mediated by different Ligand-Receptor (L-R) pairs using DeepTalk. The box plot shows the differences in L-R pair **A**. Ccl3-Ccr5, **B**. Csf1-Csf1r, **C**. Gdf15-Tgfbr2, **D**. Nrg3-Erbb4, **E**. Pdgfa-Pdgfra, **F**. Pdgfa-Pdgfrb, **G**. Pyy-Npy1r, **H**. Vegfa-Flt1, and **I**. Vip-Vipr2. The two-sided Mann-Whitney U-test is used to test the significance level. Source data are provided as a Source Data file.

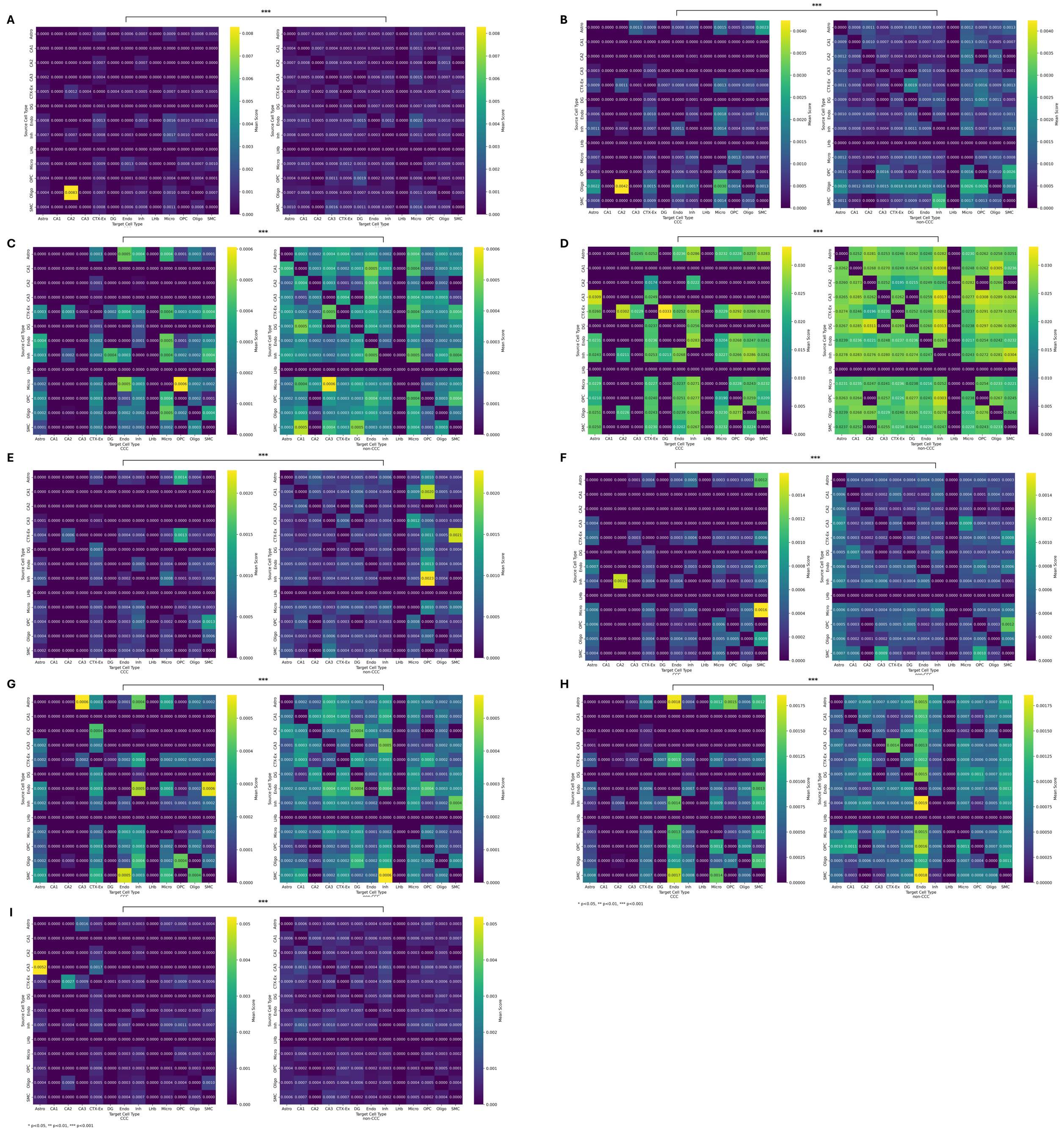

Supplementary Fig. 26 Heatmaps of the communication scores between all cell types between CCC (left) and non-CCC (right) regions mediated by different L-R pairs using DeepTalk. Heatmaps show the differences in L-R pair **A**. Ccl3-Ccr5, **B**. Csf1-Csf1r, **C**. Gdf15-Tgfbr2, **D**. Nrg3-Erbb4, **E**. Pdgfa-Pdgfra, **F**. Pdgfa-Pdgfrb, **G**. Pyy-Npy1r, **H**. Vegfa-Flt1, and **I**. Vip-Vipr2. The two-sided Mann-Whitney U-test is used to test the significance level. Source data are provided as a Source Data file.

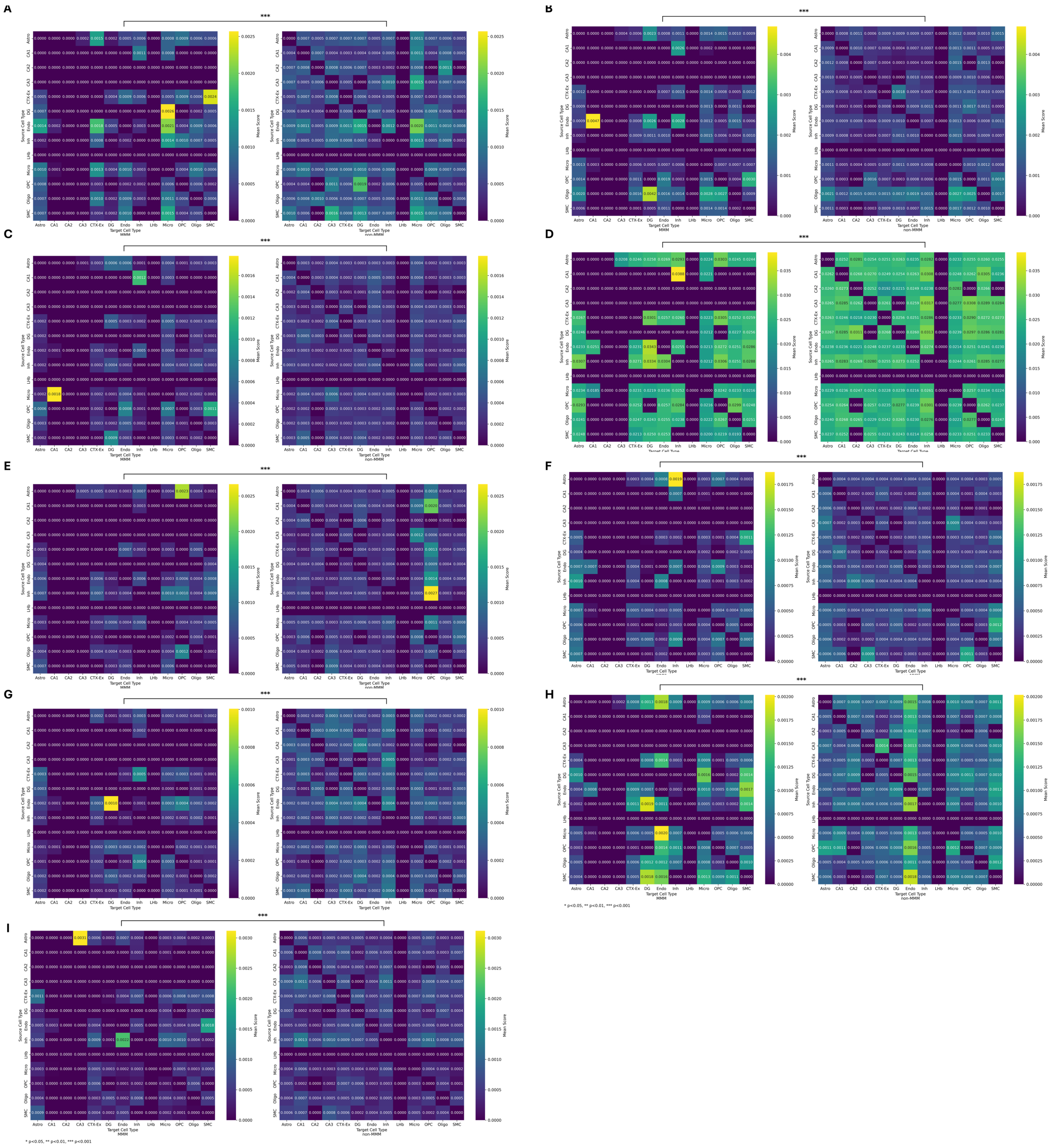

Supplementary Fig. 27 Heatmaps of the communication scores between all cell types between MMM (left) and non-MMM (right) regions mediated by different L-R pairs using DeepTalk. Heatmaps show the differences in L-R pair **A**. Ccl3-Ccr5, **B**. Csf1-Csf1r, **C**. Gdf15-Tgfbr2, **D**. Nrg3-Erbb4, **E**. Pdgfa-Pdgfra, **F**. Pdgfa-Pdgfrb, **G**. Pyy-Npy1r, **H**. Vegfa-Flt1, and **I**. Vip-Vipr2. The two-sided Mann-Whitney U-test is used to test the significance level. Source data are provided as a Source Data file.

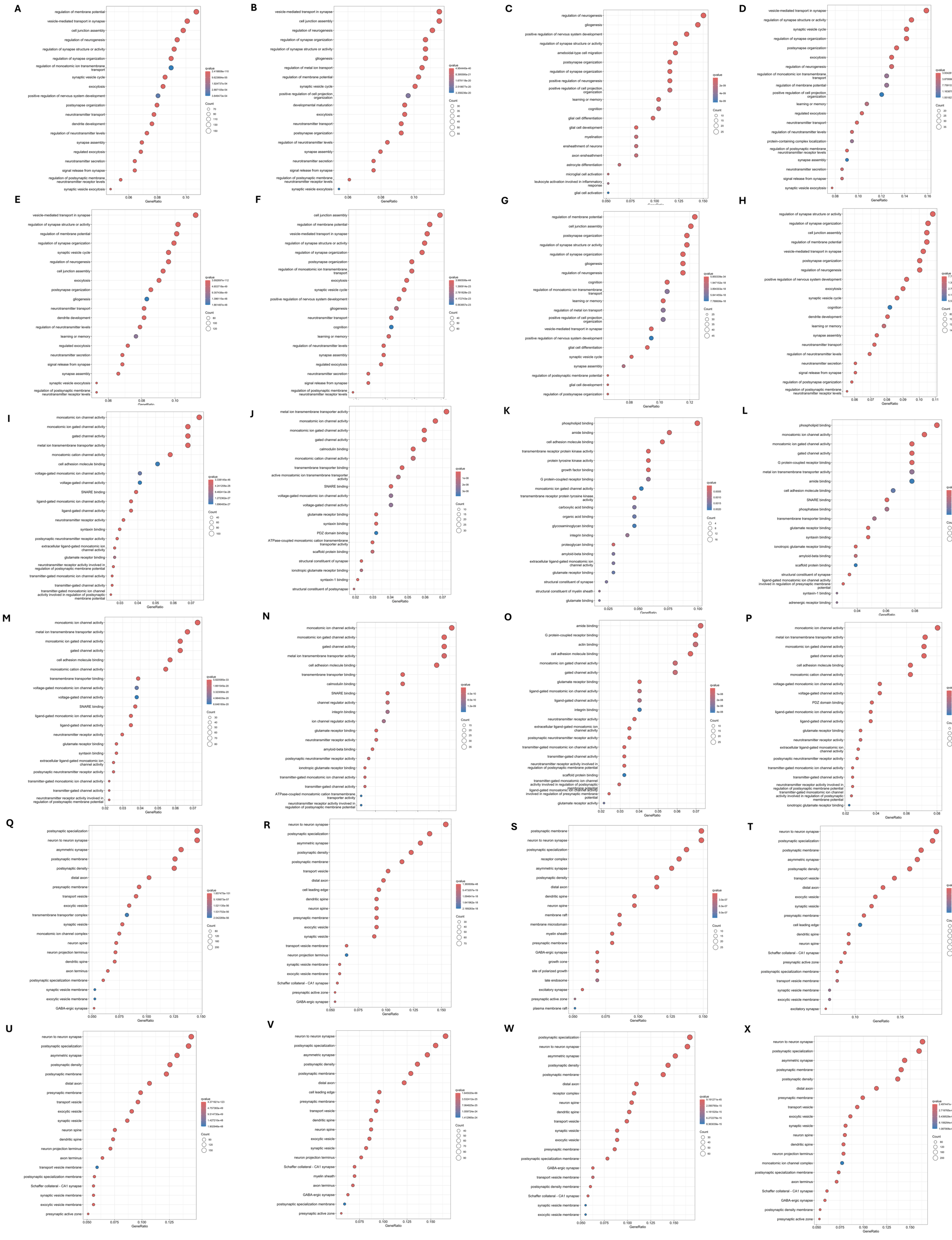
A
B
C
D
E
F
G
H
I
J
K
L
M
N
O
P
Q
R
S
T
U
V
W
X

Supplementary Fig. 28 GO enrichment analysis results for DEGs in size-3 CC motif regions. GO enrichment analysis on Biological Process results in eight-month-old samples for CC motifs **A.** 'CCC', **B.** 'CCM'**, C.** 'CMM', and **D.** 'MMM'. GO enrichment analysis on Biological Process results in thirteen-month-old samples for CC motifs **E.** 'CCC', **F.** 'CCM', **G.** 'CMM'**,** and **H.** 'MMM'. GO enrichment analysis on Molecular Function results in eight-month-old samples for CC motifs **I.** 'CCC', **J.** 'CCM'**, K.** 'CMM', and **L.** 'MMM'. GO enrichment analysis on Molecular Function results in thirteen-month-old samples for CC motifs **M.** 'CCC', **N.** 'CCM'**, O.** 'CMM', and **P.** 'MMM'. GO enrichment analysis on Cellular Component results in eight-month-old samples for CC motifs **Q.** 'CCC', **R.** 'CCM'**, S.** 'CMM', and **T.** 'MMM'. GO enrichment analysis on Cellular Component results in thirteen-month-old samples for CC motifs **U.** 'CCC', **V.** 'CCM'**, W.** 'CMM', and **X.** 'MMM'.

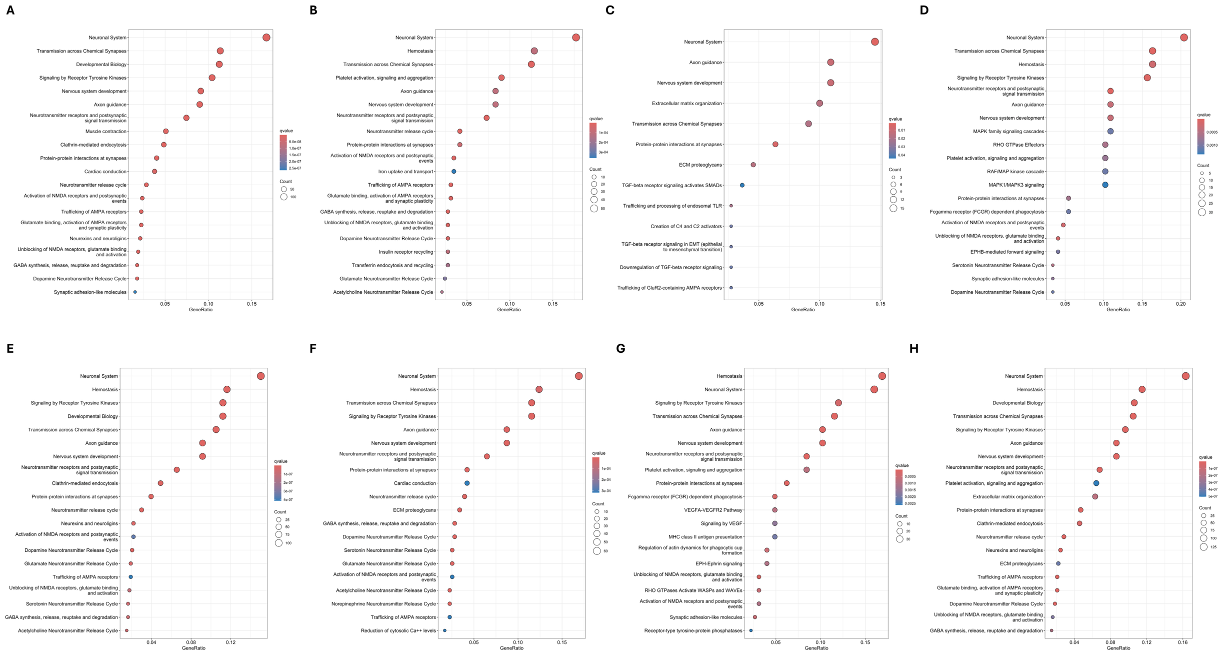

Supplementary Fig. 29 Pathway enrichment analysis results for DEGs in size-3 CC motif regions. Pathway enrichment analysis results in eight-month-old samples for CC motifs **A.** ‘CCC’, **B.** ‘CCM’**, C.** ‘CMM’, and **D.** ‘MMM’. Pathway enrichment analysis results in thirteen-month-old samples for CC motifs **E.** ‘CCC’, **F.** ‘CCM’, **G.** ‘CMM’**,** and **H.** ‘MMM’.

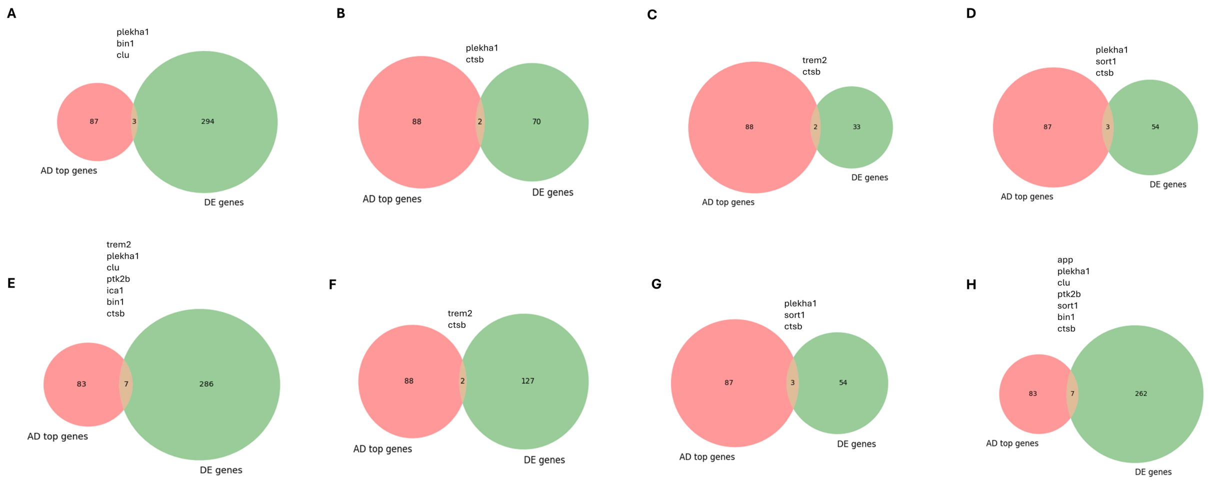

Supplementary Fig. 30 The Venn diagrams of top genes by large-scale Alzheimer's disease GWAS analysis and DEGs in CC motif regions, including gene overlaps for motif 'CCC' in **A.** eight-month-old and **B.** 13-month-old samples; motif 'CCM' in **C.** eight-month-old and **D.** thirteen-month-old samples; motif 'CMM' in **E.** eight-month-old and **F.** thirteen-month-old samples; motif 'MMM' in **G.** eight-month-old and **H.** thirteen-month-old samples.

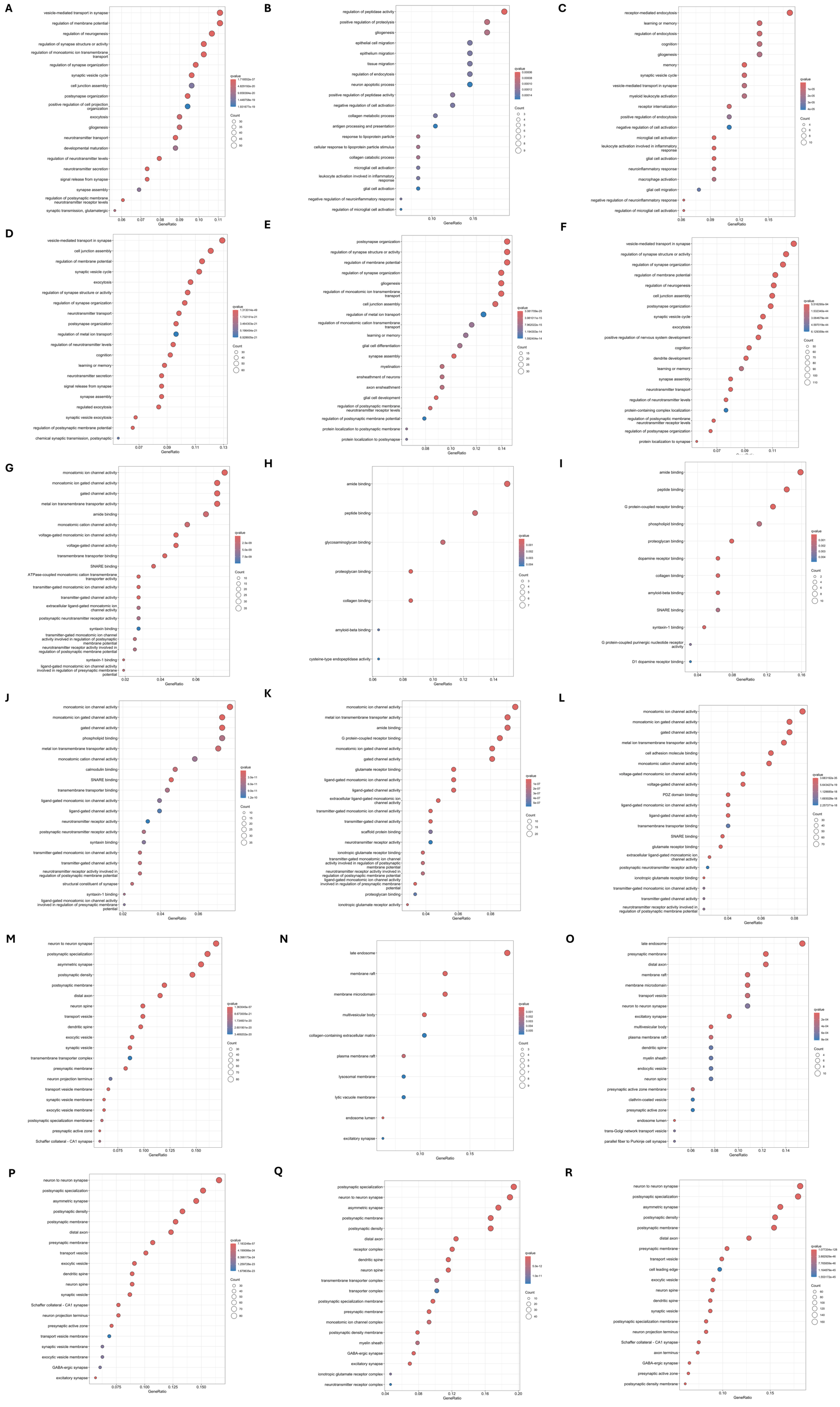
A
vesicle-mediated transport in synapse
regulation of membrane potential
regulation of neurogenesis
regulation of synapse structure or activity
regulation of monoatomic ion transmembrane transport
regulation of synapse organization
synaptic vesicle cycle
cell junction assembly
postsynapse organization
positive regulation of cell projection organization
exocytosis
gliogenesis
neurotransmitter transport
developmental maturation
regulation of neurotransmitter levels
neurotransmitter secretion
signal release from synapse
synapse assembly
regulation of postsynaptic membrane neurotransmitter receptor levels
synaptic transmission, glutamatergic
GeneRatio
qvalue
Count
B
regulation of peptidase activity
positive regulation of proteolysis
gliogenesis
epithelial cell migration
epithelium migration
tissue migration
regulation of endocytosis
neuron apoptotic process
positive regulation of peptidase activity
negative regulation of cell activation
collagen metabolic process
antigen processing and presentation
response to lipoprotein particle
cellular response to lipoprotein particle stimulus
collagen catabolic process
microglial cell activation
leukocyte activation involved in inflammatory response
glial cell activation
negative regulation of neuroinflammatory response
regulation of microglial cell activation
GeneRatio
C
receptor-mediated endocytosis
learning or memory
regulation of endocytosis
cognition
gliogenesis
memory
synaptic vesicle cycle
vesicle-mediated transport in synapse
myeloid leukocyte activation
receptor internalization
positive regulation of endocytosis
negative regulation of cell activation
microglial cell activation
leukocyte activation involved in inflammatory response
glial cell activation
neuroinflammatory response
macrophage activation
glial cell migration
negative regulation of neuroinflammatory response
regulation of microglial cell activation
GeneRatio
D
vesicle-mediated transport in synapse
cell junction assembly
regulation of membrane potential
synaptic vesicle cycle
exocytosis
regulation of synapse structure or activity
regulation of synapse organization
neurotransmitter transport
postsynapse organization
regulation of metal ion transport
regulation of neurotransmitter levels
cognition
learning or memory
neurotransmitter secretion
signal release from synapse
synapse assembly
regulated exocytosis
synaptic vesicle exocytosis
regulation of postsynaptic membrane potential
chemical synaptic transmission, postsynaptic
GeneRatio
E
postsynapse organization
regulation of synapse structure or activity
regulation of membrane potential
regulation of synapse organization
gliogenesis
regulation of monoatomic ion transmembrane transport
cell junction assembly
regulation of metal ion transport
regulation of monoatomic cation transmembrane transport
learning or memory
glial cell differentiation
synapse assembly
myelination
ensheathment of neurons
axon ensheathment
glial cell development
regulation of postsynaptic membrane neurotransmitter receptor levels
regulation of postsynaptic membrane potential
protein localization to postsynaptic membrane
protein localization to postsynapse
GeneRatio
F
vesicle-mediated transport in synapse
regulation of synapse structure or activity
regulation of synapse organization
regulation of membrane potential
regulation of neurogenesis
cell junction assembly
postsynapse organization
synaptic vesicle cycle
exocytosis
positive regulation of nervous system development
cognition
dendrite development
learning or memory
synapse assembly
neurotransmitter transport
regulation of neurotransmitter levels
protein-containing complex localization
regulation of postsynaptic membrane neurotransmitter receptor levels
regulation of postsynapse organization
protein localization to synapse
G
monoatomic ion channel activity
monoatomic ion gated channel activity
gated channel activity
metal ion transmembrane transporter activity
amide binding
monoatomic cation channel activity
voltage-gated monoatomic ion channel activity
voltage-gated channel activity
transmembrane transporter binding
SNARE binding
ATPase-coupled monoatomic cation transmembrane transporter activity
transmitter-gated monoatomic ion channel activity
transmitter-gated channel activity
extracellular ligand-gated monoatomic ion channel activity
postsynaptic neurotransmitter receptor activity
syntaxin binding
transmitter-gated monoatomic ion channel activity involved in regulation of postsynaptic membrane potential
neurotransmitter receptor activity involved in regulation of postsynaptic membrane potential
syntaxin-1 binding
ligand-gated monoatomic ion channel activity involved in regulation of presynaptic membrane potential
GeneRatio
H
amide binding
peptide binding
glycosaminoglycan binding
proteoglycan binding
collagen binding
amyloid-beta binding
cysteine-type endopeptidase activity
GeneRatio
I
amide binding
peptide binding
G protein-coupled receptor binding
phospholipid binding
proteoglycan binding
dopamine receptor binding
collagen binding
amyloid-beta binding
SNARE binding
syntaxin-1 binding
G protein-coupled purinergic nucleotide receptor activity
D1 dopamine receptor binding
GeneRatio
J
monoatomic ion channel activity
monoatomic ion gated channel activity
gated channel activity
phospholipid binding
metal ion transmembrane transporter activity
monoatomic cation channel activity
calmodulin binding
SNARE binding
transmembrane transporter binding
ligand-gated monoatomic ion channel activity
ligand-gated channel activity
neurotransmitter receptor activity
postsynaptic neurotransmitter receptor activity
syntaxin binding
transmitter-gated monoatomic ion channel activity
transmitter-gated channel activity
neurotransmitter receptor activity involved in regulation of postsynaptic membrane potential
structural constituent of synapse
syntaxin-1 binding
ligand-gated monoatomic ion channel activity involved in regulation of presynaptic membrane potential
GeneRatio
K
monoatomic ion channel activity
metal ion transmembrane transporter activity
amide binding
G protein-coupled receptor binding
monoatomic ion gated channel activity
gated channel activity
glutamate receptor binding
ligand-gated monoatomic ion channel activity
ligand-gated channel activity
extracellular ligand-gated monoatomic ion channel activity
transmitter-gated monoatomic ion channel activity
transmitter-gated channel activity
scaffold protein binding
neurotransmitter receptor activity
ionotropic glutamate receptor binding
transmitter-gated monoatomic ion channel activity involved in regulation of postsynaptic membrane potential
neurotransmitter receptor activity involved in regulation of postsynaptic membrane potential
ligand-gated monoatomic ion channel activity involved in regulation of presynaptic membrane potential
proteoglycan binding
ionotropic glutamate receptor activity
GeneRatio
L
monoatomic ion channel activity
monoatomic ion gated channel activity
gated channel activity
metal ion transmembrane transporter activity
cell adhesion molecule binding
monoatomic cation channel activity
voltage-gated monoatomic ion channel activity
voltage-gated channel activity
PDZ domain binding
ligand-gated monoatomic ion channel activity
ligand-gated channel activity
transmembrane transporter binding
SNARE binding
glutamate receptor binding
extracellular ligand-gated monoatomic ion channel activity
postsynaptic neurotransmitter receptor activity
ionotropic glutamate receptor binding
transmitter-gated monoatomic ion channel activity
transmitter-gated channel activity
neurotransmitter receptor activity involved in regulation of postsynaptic membrane potential
GeneRatio
M
neuron to neuron synapse
postsynaptic specialization
asymmetric synapse
postsynaptic density
postsynaptic membrane
distal axon
neuron spine
transport vesicle
dendritic spine
exocytic vesicle
synaptic vesicle
transmembrane transporter complex
presynaptic membrane
neuron projection terminus
transport vesicle membrane
synaptic vesicle membrane
exocytic vesicle membrane
postsynaptic specialization membrane
presynaptic active zone
Schaffer collateral - CA1 synapse
GeneRatio
N
late endosome
membrane raft
membrane microdomain
multivesicular body
collagen-containing extracellular matrix
plasma membrane raft
lysosomal membrane
lytic vacuole membrane
endosome lumen
excitatory synapse
GeneRatio
O
late endosome
presynaptic membrane
distal axon
membrane raft
membrane microdomain
transport vesicle
neuron to neuron synapse
excitatory synapse
multivesicular body
plasma membrane raft
dendritic spine
myelin sheath
endocytic vesicle
neuron spine
presynaptic active zone membrane
clathrin-coated vesicle
presynaptic active zone
endosome lumen
trans-Golgi network transport vesicle
parallel fiber to Purkinje cell synapse
GeneRatio
P
neuron to neuron synapse
postsynaptic specialization
asymmetric synapse
postsynaptic density
postsynaptic membrane
distal axon
presynaptic membrane
transport vesicle
exocytic vesicle
dendritic spine
neuron spine
synaptic vesicle
Schaffer collateral - CA1 synapse
neuron projection terminus
presynaptic active zone
transport vesicle membrane
synaptic vesicle membrane
exocytic vesicle membrane
GABA-ergic synapse
excitatory synapse
GeneRatio
Q
postsynaptic specialization
neuron to neuron synapse
asymmetric synapse
postsynaptic membrane
postsynaptic density
distal axon
receptor complex
dendritic spine
neuron spine
transmembrane transporter complex
transporter complex
postsynaptic specialization membrane
presynaptic membrane
monoatomic ion channel complex
postsynaptic density membrane
myelin sheath
GABA-ergic synapse
excitatory synapse
ionotropic glutamate receptor complex
neurotransmitter receptor complex
GeneRatio
R
neuron to neuron synapse
postsynaptic specialization
asymmetric synapse
postsynaptic density
postsynaptic membrane
distal axon
presynaptic membrane
transport vesicle
cell leading edge
exocytic vesicle
neuron spine
dendritic spine
synaptic vesicle
postsynaptic specialization membrane
neuron projection terminus
Schaffer collateral - CA1 synapse
axon terminus
GABA-ergic synapse
presynaptic active zone
postsynaptic density membrane
GeneRatio

Supplementary Fig. 31 GO enrichment analysis results for DEGs in size-4 CC motif regions. GO enrichment analysis on Biological Process results in eight-month-old samples for motifs **A**. ‘CCCM’, **B**. ‘MMMC’, and **C**. ‘MMMM’. GO enrichment analysis on Biological Process results in thirteen-month-old samples for motifs **D**. ‘CCCM’, **E**. ‘MMMC’, and **F**. ‘MMMM’. GO enrichment analysis on Molecular Function results in eight-month-old samples for motifs **G** ‘CCCM’, **H**. ‘MMMC’, and **I**. ‘MMMM’. GO enrichment analysis on Molecular Function results in thirteen-month-old samples for motifs **J**. ‘CCCM’, **K**. ‘MMMC’, and **L**. ‘MMMM’. GO enrichment analysis on Cellular Component results in eight-month-old samples for motifs **M**. ‘CCCM’, **N**. ‘MMMC’, and **O**. ‘MMMM’. GO enrichment analysis on Cellular Component results in thirteen-month-old samples for motifs **P**. ‘CCCM’, **Q**. ‘MMMC’, and **R**. ‘MMMM’.

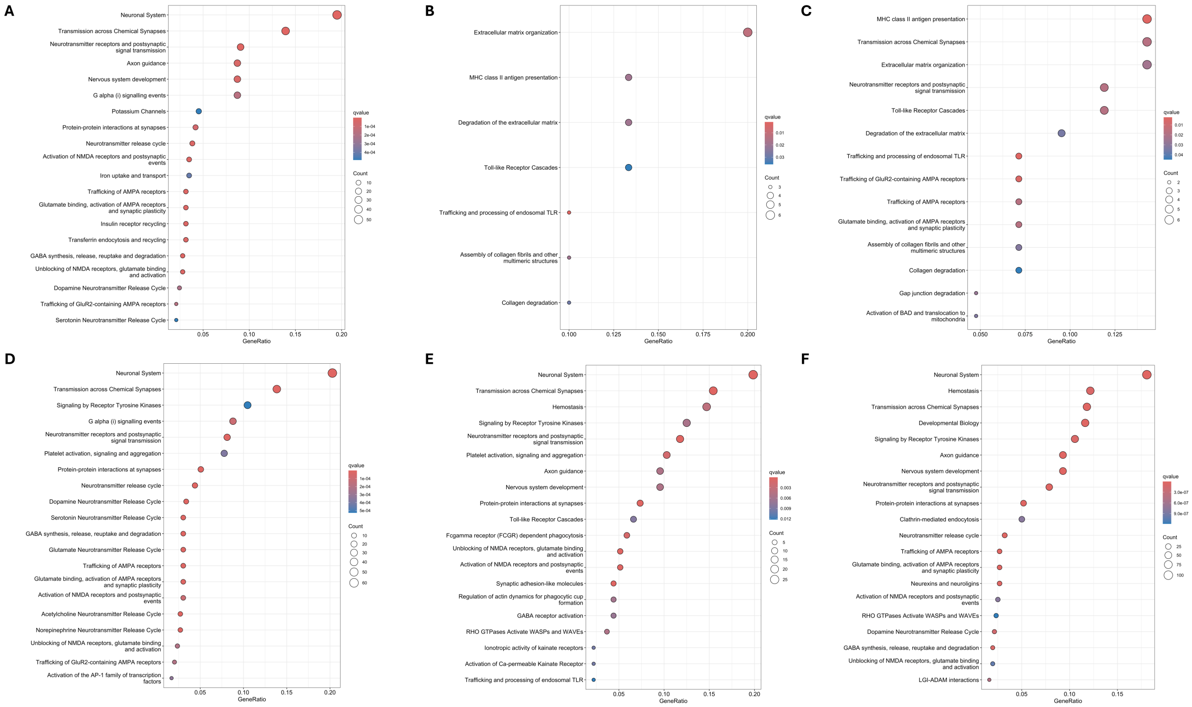

Supplementary Fig. 32 Pathway enrichment analysis results for DEGs in size-4 CC motif regions. Pathway enrichment analysis results in eight-month-old samples for CC motifs **A.** ‘CCCM’, **B.** ‘MMMC’, and **C.** ‘MMMM’. Pathway enrichment analysis results in thirteen-month-old samples for CC motifs **D.** ‘CCCM’, **E.** ‘MMMC’, and **F.** ‘MMMM’.

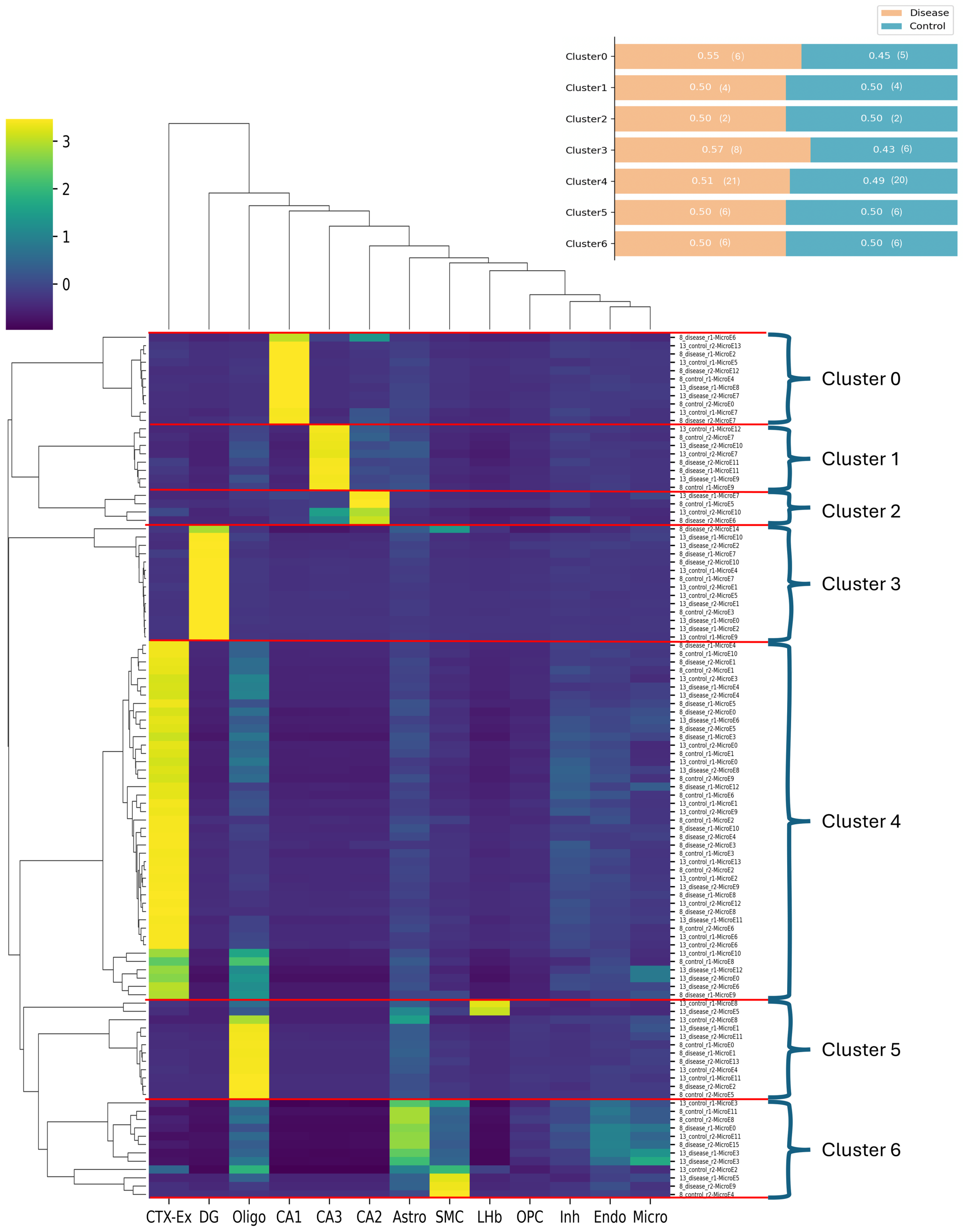

Supplementary Fig. 33 Hierarchical clustering on all Banksy-defined microenvironments on each of 8 Alzheimer's disease samples. Each sample identified 13 microenvironments. The Y-axis represents 7 clusters shared in all microenvironments of AD samples. The X-

axis represents cell type composition. The top right corner shows the proportion bar plot of disease and control samples in each cluster. The number in the parentheses indicates the number of samples in the disease and control groups. These clusters do not directly differentiate AD and non-AD samples. Source data are provided as a Source Data file.

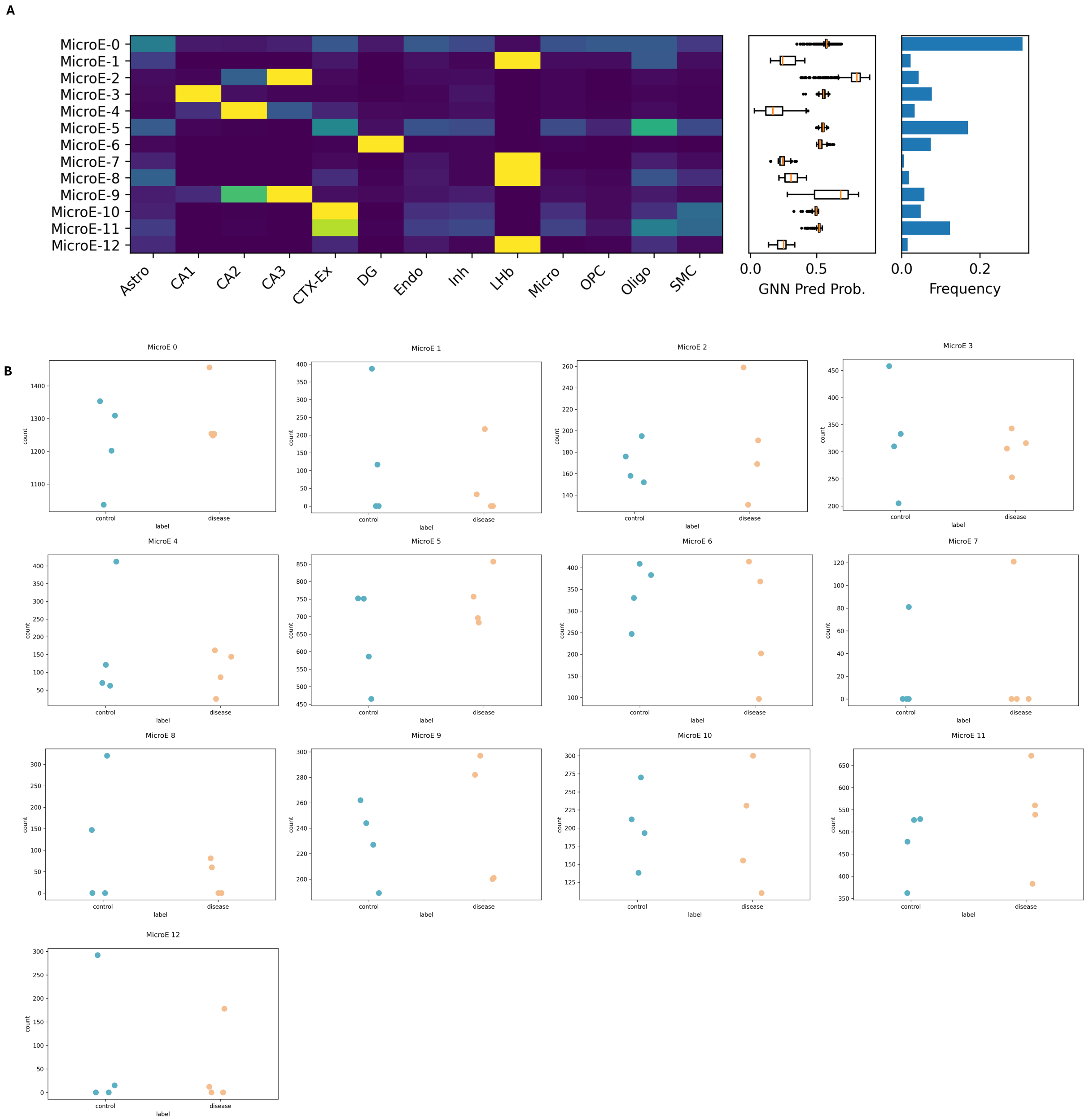

Supplementary Fig. 34 A left: Hierarchical clustering on embeddings learned from SPACE-GM on Alzheimer's disease samples. Each sample identified 13 microenvironments. The y-axis represents 13 identified microenvironments. The x-axis represents cell type composition. **B**. Jitter plots of the number of microenvironments in AD control and disease samples. None of the plots shows significance in the student t-test, demonstrating these clusters do not directly differentiate AD and non-AD samples. Source data are provided as a Source Data file.

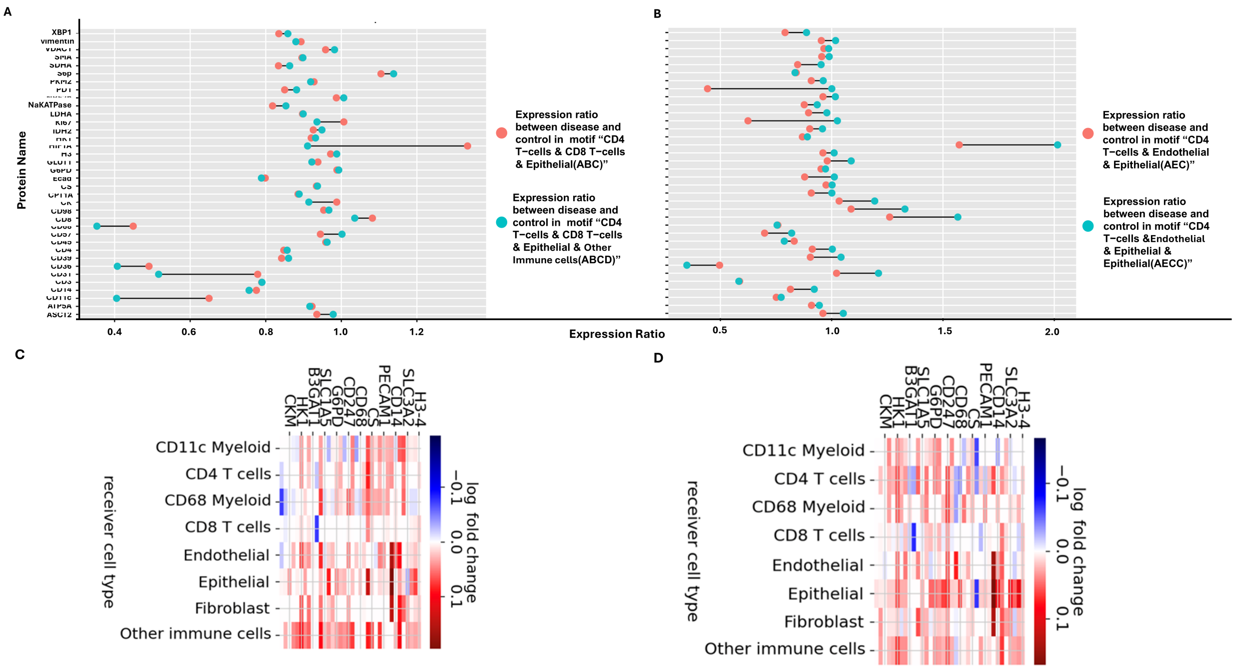

Supplementary Fig. 35 Dumbbell plot of expression ratio between disease and control samples in **A.** Shifted Interaction Motif and **B**. Homeostatic Interaction Motif. Red nodes denote the expression ratio in the size-3 motifs, and blue nodes denote its successor size-4 motifs. Sender effect on CD4 T-cells in **C**. Colon Carcinoma samples and **D**. Healthy control sample by NCEM gene-wise analysis. Source data are provided as a Source Data file.

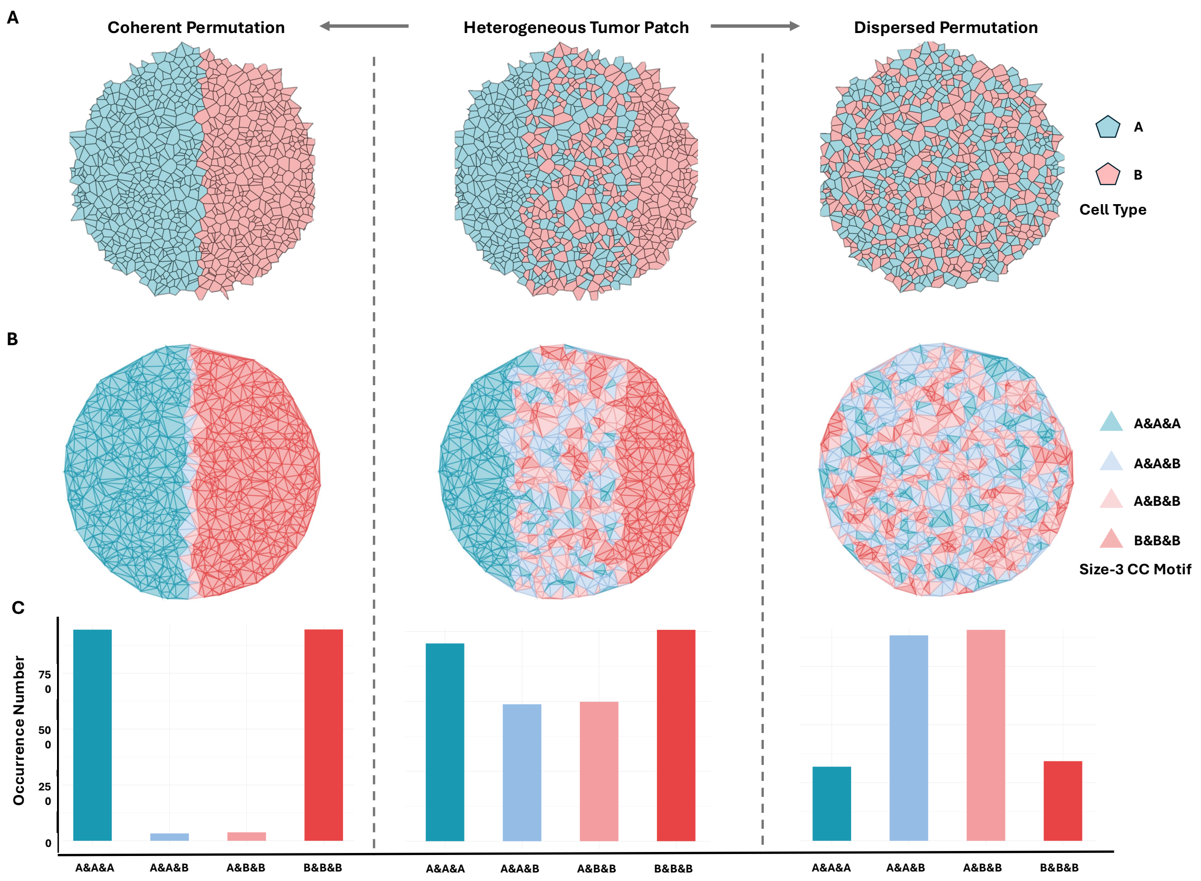

Supplementary Fig. 36 A demo system describes the dispersed and coherent cell organizations by the abundance of CC motifs. **A**. The spatial distribution of cell type A (Blue) and B (Red) in different cell organizations. **B**. The distribution of size-3 CC motif “A&A&A”,” A&A&B”,” A&B&B”, and ”B&B&B” across different cell organizations. **C**. The abundance of size-3 CC motifs “A&A&A”,” A&A&B”,” A&B&B”, and” B&B&B” as occurrence numbers across different cell organizations.

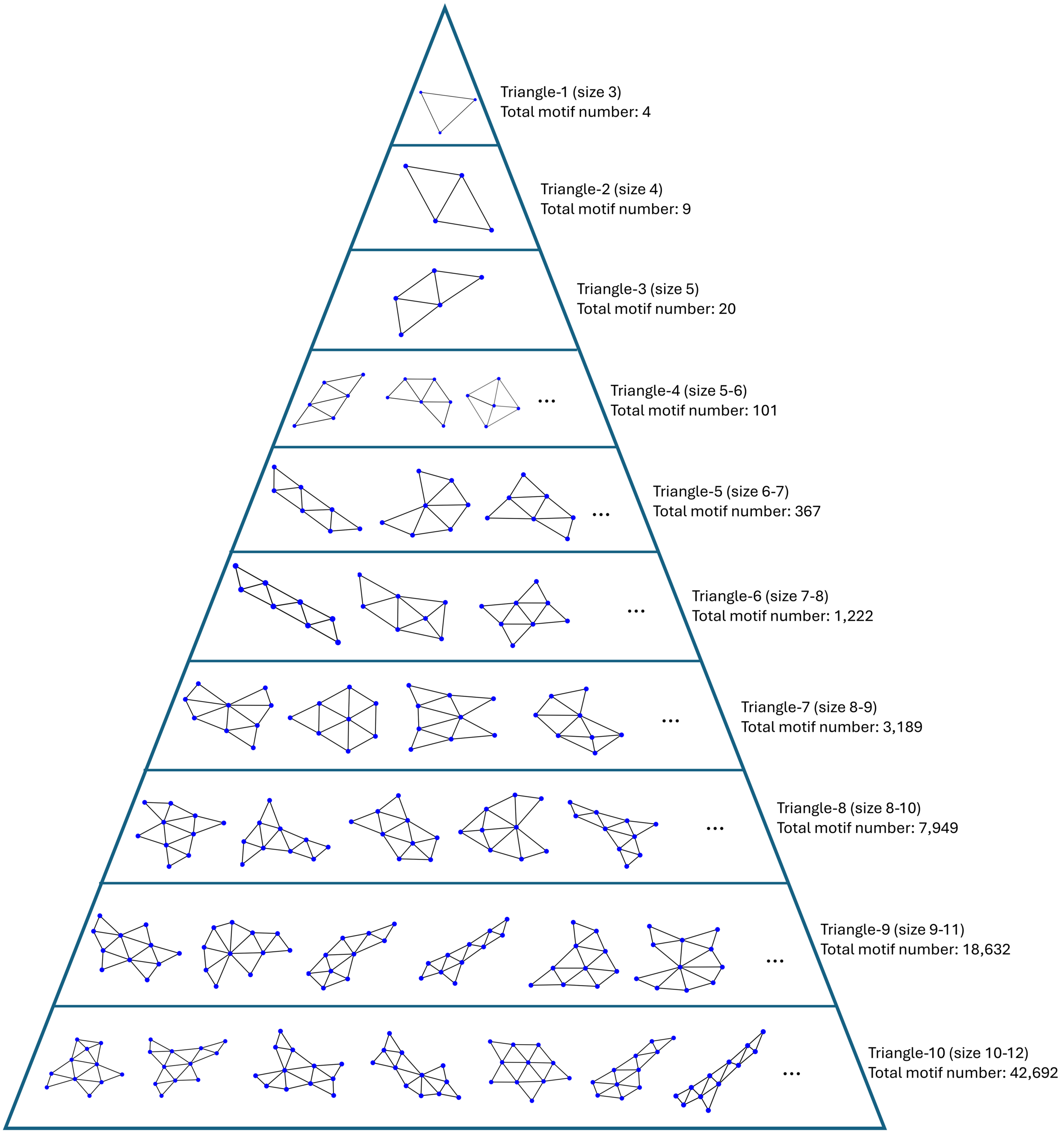

Supplementary Fig. 37 Topology of CC motifs composed of triangles from Triangle-1 to Triangle-10. Total motif number denotes the number of unique CC motifs with cell types 'CTX-Ex' and 'Microglia' as node types in the Alzheimer's disease 13-month-old replicate-1 sample.

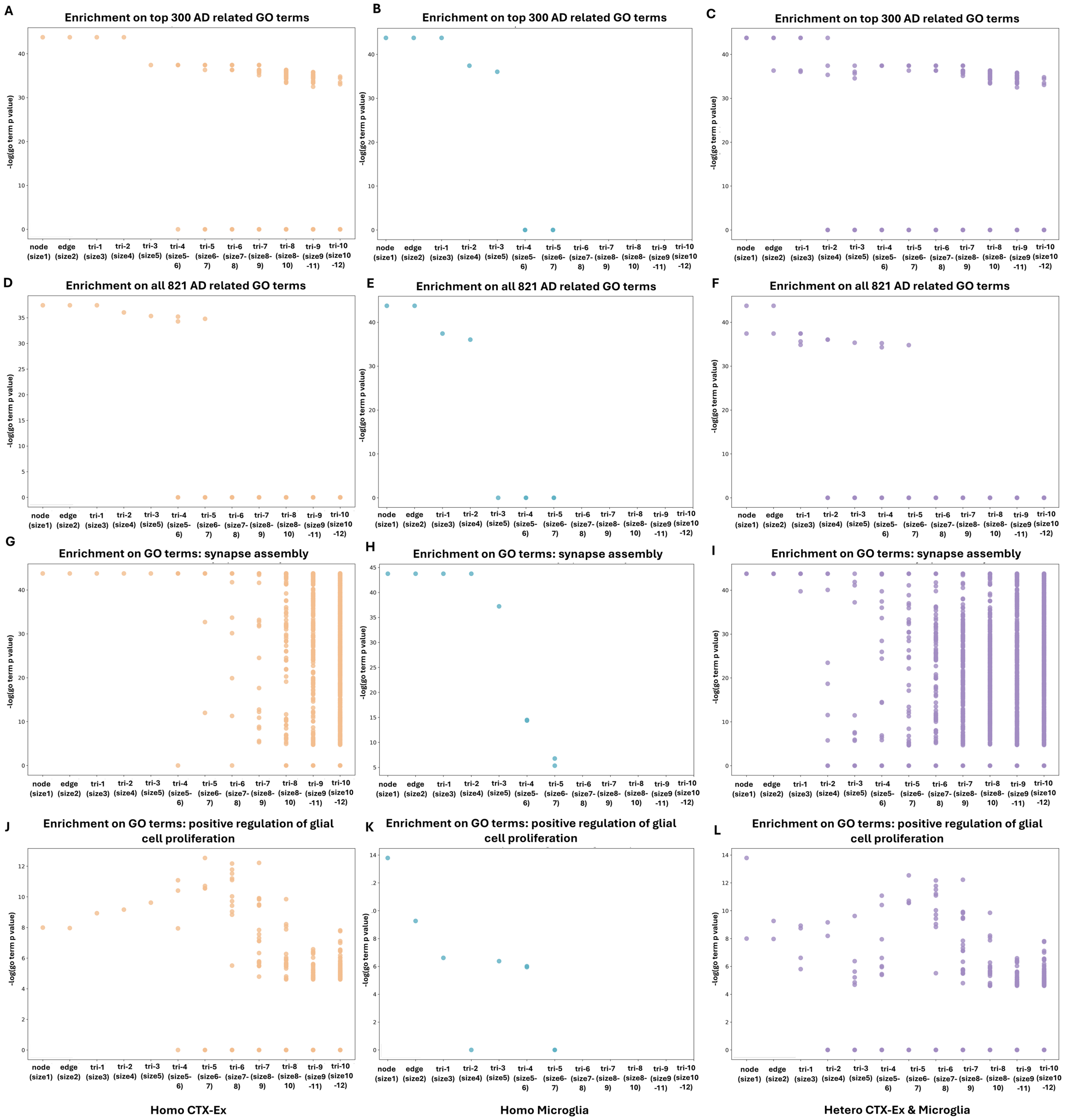

Supplementary Fig. 38 Assessment of biological relevance using GO enrichment analysis of DEGs within and out of multiple-size CC motifs. The Y-axis represents the enrichment of top 300 AD-related GO terms of DEGs from CC motifs in Homogenous CTX-Ex (**A**), Homogenous Microglia (**B**), and Heterogenous CTX-Ex and Microglia (**C**). Enrichment on all 821 AD-related GO terms of DEGs from motifs in Homogenous CTX-Ex (**D**), Homogenous Microglia (**E**), and Heterogenous CTX-Ex and Microglia (**F**). Enrichment on GO term ‘Synapse Assembly’ of DEGs from motifs in Homogenous CTX-Ex (**G**), Homogenous Microglia (**H**), and Heterogenous CTX-Ex and Microglia (**I**). Enrichment on GO term ‘Positive Regulation of Glial Cell Proliferation’ of DEGs from motifs in Homogenous CTX-Ex (**J**), Homogenous Microglia (**K**), and Heterogenous CTX-Ex and Microglia (**L**). The X-axis represents the motifs increasing in size. Each data point

represents the -log(p value) of enrichment on specific GO terms from DEGs within and out of motif regions. Here AD-related GO terms represent the intersected significant GO terms among size-3 motifs – ‘CCC’, ‘CCM’, ‘CMM’, and ‘MMM’ in 13-month-old disease samples in AD research. Source data are provided as a Source Data file.

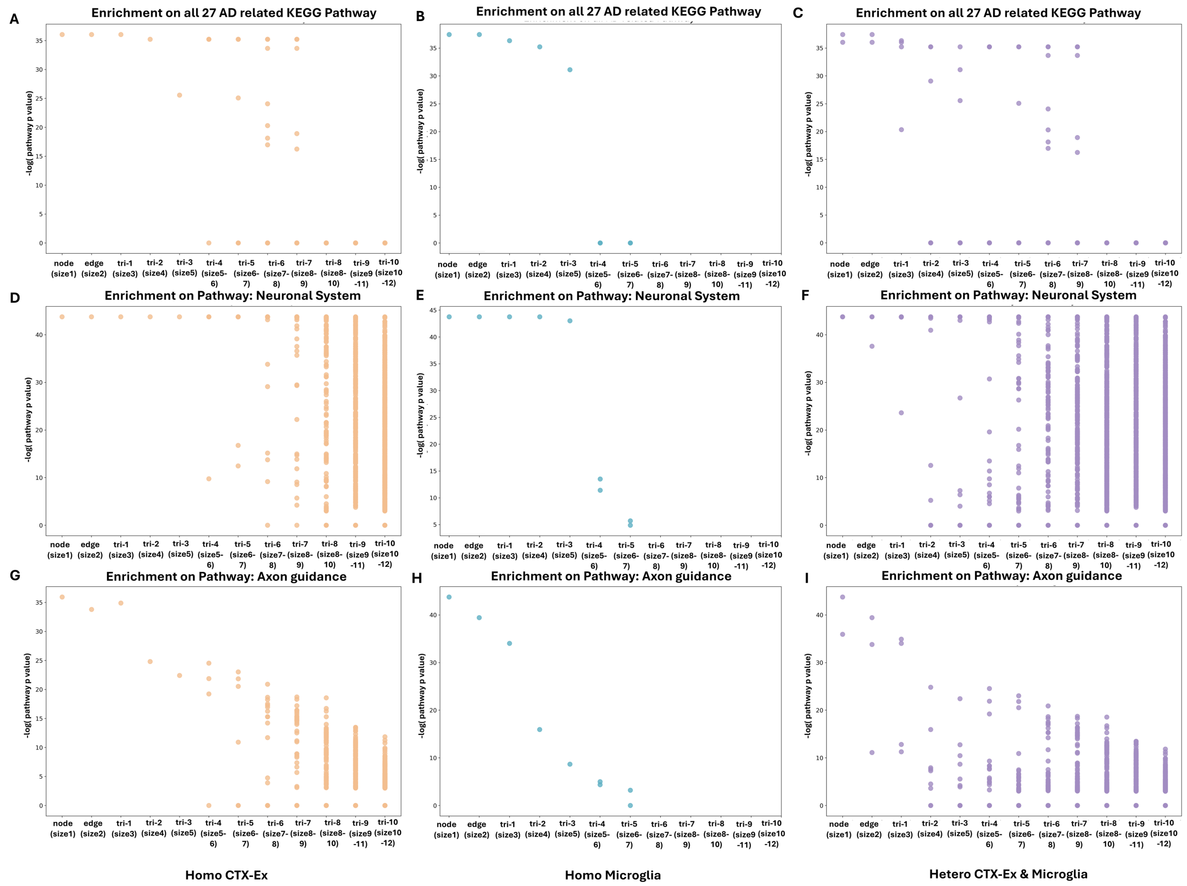

Supplementary Fig.39 Assessment of biological relevance using KEGG Pathway enrichment analysis of DEGs within and out of multiple size CC motifs. The Y-axis represents the enrichment of all 27 AD-related pathways of DEGs from CC motifs in Homogenous CTX-Ex (**A**), Homogenous Microglia (**B**), and Heterogenous CTX-Ex and Microglia(**C**). Enrichment on Pathway 'Neuronal System' of DEGs from motifs in Homogenous CTX-Ex (**D**), Homogenous Microglia (**E**), and Heterogenous CTX-Ex and Microglia **(F)**. Enrichment on Pathway 'Axon guidance' of DEGs from motifs in Homogenous CTX-Ex (**G**), Homogenous Microglia (**H)**, and Heterogenous CTX-Ex and Microglia (**I**). The X-axis represents the motifs increasing in size. Each data point represents the -log(p value) of enrichment on specific Pathways from DEGs within and out of motif regions. Here AD-related Pathways represent the intersected significant Pathways among size-3 motifs – 'CCC', 'CCM', 'CMM', and 'MMM' in 13-month-old disease samples in AD research. Source data are provided as a Source Data file.

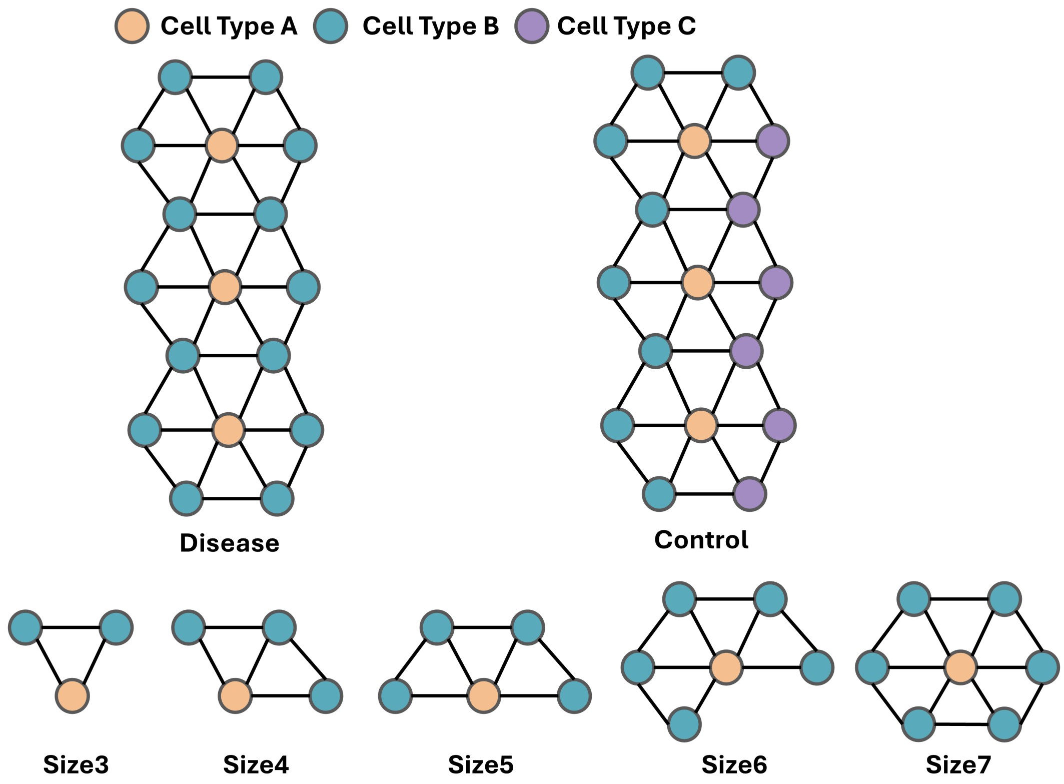

Supplementary Fig. 40 Simulated triangulated graphs differ in cell type distribution in disease and control conditions. Specific size-3 to size-7 CC motifs are simulated to check their abundance in case and control conditions

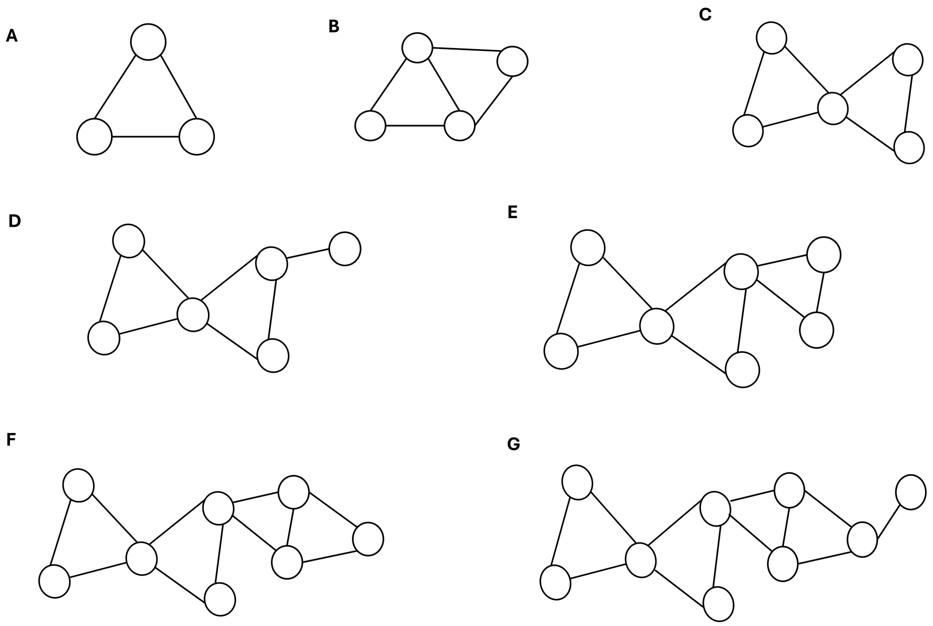

Supplementary Fig. 41 Seven CC motifs in different topologies used in the training dataset of TrimNN, including **A.** size-3. **B.** size-4. **C.** size-5. **D.** size6. **E.** size-7. **F.** size-8. **G.** size-9.

Supplementary Notes

1. Analysis of CC motif 'CCC' and pattern growth using TrimNN in AD study

Similar to analyzing CC motif 'MMM', we also analyzed CC motif 'CCC'. Comparing 'CCC' motif-enriched and complementary regions, differentially expressed genes (DEGs) were identified as significant (*p*-value<0.05) using DESeq2, including *Plekha1*, *Bin1*, *Clu*, *Ctsb*, *Sort1* in eight-month-old samples, and *Trem2*, *App*, *Ptk2b*, *Bin1*, *Ctsb* in thirteen-month-old samples. On DEGs in thirteen-month-old samples, Gene Ontology (GO) enrichment analysis showed significant vesicle-mediated transport in synapse (*q*-value = 9.09e-47), synaptic vesicle cycle (*q*-value 6.87e-41), regulation of synapse structure (*q*-value = 7.79e-32), and neural transmitter transport (*q*-value 8.48e-30) (Supplementary Fig. 28E). Neural systems (*q*-value = 2.91e-20), Transmission across Chemical Synapses (*q*-value = 4.33e-16), Neurotransmitter receptors and postsynaptic signal transmission (*q*-value = 1.20e-07), Apoptosis (*q*-value = 4.62e-05), and Norepinephrine Neurotransmitter Release Cycle (*q*-value = 4.62e-05) were enriched with pathway enrichment analysis (Supplementary Fig. 29E).

Based on the identified size-3 motif 'CCC', we performed pattern growth to identify size-4 motifs using TrimNN. Among all the size-4 'CCC' expanded motifs, 'CCCM' showed the most significant difference between AD and control samples upon the motif 'CCC' with *p-value* 1.78e-09 and 5.04e-21 using Benjamini-Hochberg adjusted Fisher's exact test. The 'CCCM' motif occurred 434 and 252 times in the sum of two replicates of eight-month-old samples and 368 and 266 in the sum of two replicates of thirteen-month-old samples, respectively. Similar to size-3 analysis, CellChat identified the cell-cell communications of motif 'CCCM' and its 3-hop regions, and we found genes *GRN*, *CCL*, *VIP*, *VEGF*, *PDGF*, *PTN*, and *CXCL* are consistent with the results from the size-3 motifs. The top AD risk genes intersected with DEGs in 'CCCM' are *Ctsb* in eight-month-old samples and *Sort1*, *Bin1*, *Ctsb*, *App*, and *Ptk2b* in thirteen-month-old samples. Using these DEGs in motif 'CCCM' in thirteen-month-old samples, GO enrichment analysis (Supplementary Fig. 31D) showed that post-synapse organization (*q*-value = 7.59e-23), learning or memory (*q*-value = 2.63e-14), cognition (*q*-value = 1.93e-13), and receptor localization to synapse (*q*-value = 1.22e-15) were enriched biological processes. Also, Pathway enrichment analysis (Supplementary Fig. 32D) showed Neuronal System (*q*-value = 1.76e-08), Transmission across Chemical Synapses (*q*-value = 2.98e-06), Synaptic adhesion-like molecules (*q*-value = 8.27e-04), Neurexins and Neuroligins gene enrichment (*q*-value = 2.93e-03).

2. Revisit AD case study with Shifted Interaction Motifs and Homeostatic Interaction Motifs

After introducing the concepts of Shifted Interaction Motifs and Homeostatic Interaction Motifs, we revisited the AD case study by analyzing size-3 and their related size-4 CC

motifs with TrimNN. We performed a proportion test on size-3 motif ‘CCC’ and size-4 motif ‘CCCM’ among disease and control samples, where ‘C’ denotes cortex excitatory neuron and ‘M’ denotes microglia. All eight-month-old replicates 1 and 2 samples and thirteen-month-old replicates 1 and 2 samples obtained significant results with *p*-values 1.87E-05, 1.32e-06, 1.19E-15, and 5.84E-12, respectively. Meanwhile, on size-3 motif ‘MMM’ and size-4 motif ‘MMMC’ (*p*-values 0.006913, 1, 1, and 1), and size-3 motif ‘MMM’ and size motif ‘MMMM’ (*p*-values 0.2359, 1, 0.1225, and 0.9093), both were not significant using proportion test in eight-month-old replicates 1 and 2 samples and thirteen-month-old replicates 1 and 2 samples (Supplementary Data 66). These results demonstrated that the transition from motif ‘CCC’ to ‘CCCM’ was aligned with Shifted Interaction Motifs, and the other two types of motifs were aligned with Homeostatic Interaction Motifs, further validating the differences between these CC motifs.

3. TrimNN analysis on size-3 and size-4 CC motifs in the colorectal carcinoma study compared to NCEM

Compared to cell type coupling analysis in NCEM, which estimates cell interactions of the effects of niche composition, CD4 T-cell type has trends to consistently couple across different cell types in colorectal samples (Fig. 5L), i.e., CD4 T-cells have top ranks (1st, 2nd, 3rd out of 8) in edge-wise analysis and it also showed that half of the effect values in NCEM were the highest (except CD8 T-cells as receiver) in CD4 T-cells sender effect. Nevertheless, the motif analysis was stable within specific cell types, leading to more consistent rankings. On the other hand, the results from NCEM appeared to have more variations among receivers for a single sender. They did not show a pattern getting higher rank values towards inner single cell types, i.e., endothelial cells as senders, they have ranks 2nd and 8th out of 8 and also rank 8th for the homogeneous endothelial cells.

NCEM showed that CD8 T-cells as senders and epithelial cells as receivers can differentiate diseases and controls (Fig. 5R). However, more details such as CC motifs can provide more insights into spatial tendencies. Targeted the size-2 couplings of CD8 T-cells and epithelial cells, only triangle patterns combining CD4 T-cells were considered significant (*p-value* 3.58e-12, Fisher’s exact test adjusting by Benjamini-Hochberg method) among all eight possible combinations in size-3 CC motifs (Supplementary Data 65). This observation demonstrated that the triangle-like size-3 CC motif of the cell types CD4 T-cells, CD8 T-cells, and Epithelial (‘ABC’) might be essential to capture the main difference between disease and control samples in colorectal carcinoma. Based on motif ‘ABC’, TrimNN with pattern growth search also identified one size-4 CC motif with a cell type Other Immune cells (other CD45+) (‘ABCD’) significant in both disease and control samples. This was also a Shifted Interaction Motif shown in Fig. 5A.

4. Analysis of the impact of increasing motif size on the discrimination between disease and control samples

Investigating large-size motifs is a challenging task as it is an emerging research area in spatial biology. As the size grows larger, the field currently lacks established approaches to perform comprehensive and rigorous computational, statistical, and biological evaluations. According to the reviewer's guidance, we comprehensively explored this question by (1) exploring the robustness simulation to size-5 to show the robustness of multi-size motifs resistant to noises in Fig. 2A-C, (2) exploring the identified size-3 motifs to subsets of size-12 (Supplementary Figs. 37) in the context of AD research ranked by significance from functional enrichment (Supplementary Figs. 38-39), (3) a specially designed system of size-7 to demo the idealized process in identifying the appropriate size of CC motifs (Supplementary Fig. 40)

4.1 Exploring up to size-5 CC motifs in robustness simulation

We hypothesize CC motifs as the countable recurring spatial patterns of various cell types are robust within noises to represent and quantify multicellular organization. Simulations were performed to mimic different levels of noises, including cell missing from the cell capture imperfection of sequencing technology (Fig. 2A), cell coordinates shifting from technological errors (Fig. 2B), and cell type misclassification from annotation errors in data analytics (Fig. 2C). The noises are poised at cell proportions of 0.01, 0.05, 0.1, 0.2, 0.3, 0.4, and 0.5 within CC motifs in size-1, size-2, size-3, size-4 and size-5 in all these three simulations.

For each motif size, we calculated the Spearman correlation between the abundance rankings of all possible motifs in the original and the noise-poised data. If the simulated noise does not change the relative abundance, CC motifs derived by the adopted Delaunay Triangulation are robust to noises, and the correlation will be high. We observed that correlation decreases linearly across all three simulation types with increasing noise and motif size. We want to mention the length of rank lists of all the possible size-1, size-2, size-3, size-4, and size-5 motifs are 13, 91, 455, 8281, and 54418, respectively. The probability of occurrences of size-1 motifs is 1/13 and 1/54418 in size-5. Larger motifs yield more enumerated patterns, making them less likely to maintain their current status in the spatial spaces. Within the same noise level, large-size motifs are relatively sensitive to noises and easily change to another motif under the influence. This may explain the phenomenon that correlation is worse in larger motif structures.

Within the updated comprehensive simulations up to size-5, the robustness of the motifs indeed has limits. In both scenarios of missing cell (Fig. 2A) and shifting cell coordinate (Fig. 2B), the correlation values remain relatively stable even under extreme noise conditions (noise in half cells with level 0.5). For cell type misclassification (Fig. 2C), the correlation values deteriorate for larger motif sizes when the noise level goes extreme.

However, such extremely high noise levels are unlikely to occur in practical scenarios in cell type annotation. Therefore, our approach is expected to be robust in practical applications.

In practice, we are at our current HPC capacity to generate systematic results in size-5 with exponential complexity. Within the linear tendencies illustrated from size-1 to size-5, we anticipate the performances in correlation getting worse with increasing motif sizes. It should not influence our current results in size-3 and size-4 CC motifs, but it is an open question for further exploration.

4.2 Exploring up to size-12 CC motifs in the context of AD research

From the perspective of statistics, very limited approaches are available to generalize statistical significance comparison across the combinatorial space in multiple sizes. We used Fisher's exact test (or the Chi-squared test) with odds ratios as the effect size within size-2 and size-3, and we are very hesitant and careful about further statistical evaluation in sizes larger than 3. Specifically, although a three-way contingency table can be analyzed using the Cochran–Mantel–Haenszel (CMH) test[1] with the effect size measured by the Mantel–Haenszel common odds ratio, there is no single statistical test that is comparable to Fisher's exact test (or the Chi-squared test) or the CMH test—that universally applies to four-way or higher-dimensional contingency tables. Furthermore, analyzing three-way or higher-order tables involves more complex hypothesis testing, as conditional and marginal associations may differ substantially[2]. Here, we tried to borrow the idea from functional Gene Ontology (GO) and pathway enrichments to perform the significance evaluation of differentially expressed genes (DEGs) from different sizes of CC motifs. We hope this evaluation can indirectly provide a fair evaluation of which size of CC motif best describes the biology from the observed data in the slides.

Given the exponential computational complexity associated with large motifs, we investigated their biological relevance by identifying biologically meaningful motifs in the Alzheimer's Disease study containing only cell types 'CTX-Ex' (cell type C) and 'Microglia' (cell type M). In the assessment, we extended our motif size up to 10 triangles (typically size 12), and benchmarked the exact occurrences of each motif using the enumerative searching approach VF2 with guaranteed accuracy. For each motif in multiple sizes, we identified its unique DEGs between cells within and out of the motif region as in the AD case study. The quality of CC motifs is indicated and compared by q values of enriched GO terms and pathways. Ranking by Cauchy meta-analysis on -log(*p*-value), the top 300 significant GO terms in the intersection of four size-3 motifs: 'CCC', 'CCM', 'CMM', and 'MMM' in 13-month-old AD disease samples were used as baseline (Supplementary Data 69). Supplementary Fig. 38 ABC illustrate their AD relevance using enriched top 300 GO terms on DEGs from CC motifs. These motifs vary from size-1 pure cell type to triangle-10 (size-12) in homogeneous CTX-Ex (Supplementary Fig. 38A), homogeneous Microglia

(Supplementary Fig. 38B), and heterogeneous combinations (Supplementary Fig. 38C). To prevent potential bias, we also performed a similar analysis using all 821 significant GO terms (Supplementary Data 70) intersection of four mentioned size-3 motifs (Supplementary Fig. 38 DEF). In both cases, we observed non-increasing trends in significance with increasing motif sizes. Motifs in smaller sizes, usually not exceeding 4, exhibited the most significant enriched results. Notably, the largest homogeneous microglia motifs were found at size 7, consistent with the observation that microglia are scatter aggregated other than widely spread CTX-Ex cells.

Besides small-size preferred trends on GO term sets, we also found some large-size motifs that have better results on specific GO terms. Some motifs are equally significant through all the sizes in 'synapse assembly'(Supplementary Fig. 38 GHI), and homogeneous CTX-EX motifs in size 6-7 have the most significant results in GO term 'positive regulation of glial cell proliferation' (Supplementary Fig. 38 JKL). In pathway enrichment analysis, we observed a similar non-increasing trend in all 27 significant pathways intersecting four mentioned size-3 motifs in the main text (Supplementary Data 71 and Supplementary Fig. 39 ABC). This trend does not hold true when going to specific pathways. Similarly, we observe the plateau of significance on 'Neuronal System' (Supplementary Fig. 39 DEF) and the descending-ascending-descending trend in the Pathway 'Axon guidance' (Supplementary Fig. 39 GHI).

4.3 Exploring a demo system up to size-7 CC motifs

We conducted a demo experiment using simulated graphs and motifs to show the idealized process in identifying the appropriate size of CC motifs (Supplementary Fig. 40). In this simulation data, we assumed that the disease and control target graphs have different distributions of cell types. We then counted the occurrences of our simulated specific size-3 to size-7 CC motifs in these two target triangulated graphs (Supplementary Data 72).

As shown in the results, these CC motifs from size-3 to size-7 exhibit varying distinctions between disease and control samples. From size-3 to size-6, the difference in occurrence numbers between the two conditions progressively increases with motif size. However, size-7 turned this trend of differences between disease and control. In general, the likelihood and frequency of motif occurrences in the target graph decrease when increasing the motif size, until a turning point is encountered to capture the largest numerical differences between two conditions. In this case, we can select size-6 demonstrating the most significant differences between the case and the control.

Within these comprehensive experiments, along with three case studies, we discuss trade-offs between large and small sizes of CC motifs in presenting the characteristics of cellular neighborhoods in spatial biology.

(i) Complex large-size CC motifs may bring better prediction power but are hard to identify.

In the case study of CRC, we confirmed that size-3 CC motifs have better AUROC than size-1 CC motifs to distinguish subtypes of CRC using the same number of features (Fig. 3A). For specific cell type combination, size-4 CC motifs can better distinguish survival than size-3 motifs, while size-2 cannot distinguish survival (Fig. 3D, 3E, and 3F). However, the number of motifs increases exponentially, which makes it more computationally expensive to identify the most overrepresented motifs in large sizes. For example, AD data used in the study has 13 cell types, which makes 13 size-1, 91 size-2, 455 size-3, 8,281 size-4, and 54,418 size-5 motifs.

(ii) Complex large-size CC motifs have similar biological interpretability with smaller-size motifs but lack an established statistical framework in scientific rigor.

From the perspective of statistics, very limited approaches are available to generalize statistical significance comparison across the combinatorial space in multiple sizes. We used Fisher's exact test (or the Chi-squared test) with odds ratios as the effect size within size-2 and size-3, and we are very hesitant and careful about further statistical evaluation in sizes larger than 3, since analyzing three-way or higher-order tables involves more complex hypothesis testing, as conditional and marginal associations may differ substantially[2]. However, we can use motif visualization, functional analysis in GO and pathway enrichment, cell-cell communication, and phenotypic analysis to easily interpret the motif results in multiple sizes. Notably, there are no limits in sizes to use these interpretation approaches on these motifs.

(iii) Applying complex large-size CC motifs needs more caution in machine learning for overfitting.

Even CC motifs can be treated as representations of characteristics of cellular neighborhood, users are supposed to be cautious about risks of overfitting when using them as features for machine learning tasks. When applying to current spatial omics datasets with a limited number of samples, the number of features as possible CC motifs in large size can often surpass the number of sample sizes. Take the same example of AD data with 13 cell types, it has 13 size-1, 91 size-2, 455 size-3, 8,281 size-4, and 54,418 size-5 motifs. Users need to be aware of selecting the appropriate number of top features to perform machine learning tasks to prevent overfitting.

In summary, we would like to emphasize that even large-size motifs reveal their values in top-down approaches. The theory and practice of finding the most appropriate size to describe the observed data in spatial biology is still in its infancy. From the limited experiments and observations, it may not have one optimal size of CC motifs uniformly describes cellular communities within the complex biological context revealed from the spatial omics. Different approaches in diverse statistics, functional analysis, and

phenotypic analysis could reveal different perspectives in different biological scenarios. We cannot give any specific recommendation with guarantees at this time, but we believe TrimNN provides an accurate and computationally feasible way to approach this challenge.

Supplementary References:

1 Mantel, N. & Haenszel, W. Statistical aspects of the analysis of data from retrospective studies of disease. *J Natl Cancer Inst* **22**, 719-748 (1959).

2 Agresti, A. *Categorical data analysis*. (John Wiley & Sons, 2013).
