# Supplementary material for: TrimNN: characterizing cellular community motifs for studying multicellular topological organization in complex tissues
Source: Nat Commun. 2025 Aug 19;16:7737. doi: 10.1038/s41467-025-63141-7 (PMC12365081; doi:10.1038/s41467-025-63141-7)
Supplement: Supplementary file 2 — Description of Additional Supplementary Files [file 41467_2025_63141_MOESM2_ESM.pdf]

Description of Additional Supplementary Files:

Supplementary Data 1 Benchmarking the performance of TrimNN, TrimNN-RGIN, and NSIC on an independent test set for subgraph matching

Supplementary Data 2 Performance comparison of TrimNN, TrimNN-RGIN, and NSIC in identifying occurrences of CC motifs

Supplementary Data 3 Performance comparison of TrimNN, TrimNN-RGIN, and NSIC in identifying ranking index of CC motifs

Supplementary Data 4 Performance comparison of adding positional encoding in TrimNN

Supplementary Data 5 Comparison the model performances by replacing GIN with GCN and GT

Supplementary Data 6 Performance comparison of TrimNN trained with full data, half of the training data, and one-quarter of the training data

Supplementary Data 7 Performance using 10 times 10-fold cross validation on the LR model with top 5, 10, 15, and 20 size-3 CC motif features, as well as CytoCommunity using reduced 29-dimensional embedding

Supplementary Data 8 Classification using Logistics Regression with different top ranking motifs from TrimNN

Supplementary Data 9 Classification using Random Forest with different top ranking CC motifs from TrimNN

Supplementary Data 10 Classification using Support Vector Machine with different top ranking CC motifs from TrimNN

Supplementary Data 11 Performance comparison to LR model using top 29 TrimNN motifs, CytoCommunity and SPACE-GM

Supplementary Data 12 The size2, 3, 4 CC motifs with significant Cox p-value in survival curve

Supplementary Data 13 P value of node occurrence numbers of cellular community among DII and CLR samples in CRC Codex dataset

Supplementary Data 14 P value of edge occurrence numbers of cellular community among DII and CLR samples in CRC Codex dataset

Supplementary Data 15 P value of triangle occurrence numbers of cellular community among DII and CLR samples in CRC Codex dataset

Supplementary Data 16 The occurrence number of top size-4 motifs in CRC Codex cellular community

Supplementary Data 17 P value of 'CTX-Ex' and 'Microglia' related node occurrence numbers of cellular community in AD eight-month replicate 1 sample

Supplementary Data 18 P value of 'CTX-Ex' and 'Microglia' related edge occurrence numbers of cellular community in AD eight-month replicate 1 sample

Supplementary Data 19 P value of 'CTX-Ex' and 'Microglia' related triangle occurrence numbers of cellular community in AD eight-month replicate 1 sample

Supplementary Data 20 P value of 'CTX-Ex' and 'Microglia' related node occurrence numbers of cellular community in AD eight-month replicate 2 sample

Supplementary Data 21 P value of 'CTX-Ex' and 'Microglia' related edge occurrence numbers of cellular community in AD eight-month replicate 2 sample

Supplementary Data 22 P value of 'CTX-Ex' and 'Microglia' related triangle occurrence numbers of cellular community in AD eight-month replicate 2 sample

Supplementary Data 23 P value of 'CTX-Ex' and 'Microglia' related node occurrence numbers of cellular community in AD thirteen-month replicate 1 sample

Supplementary Data 24 P value of 'CTX-Ex' and 'Microglia' related edge occurrence numbers of cellular community in AD thirteen-month replicate 1 sample

Supplementary Data 25 P value of 'CTX-Ex' and 'Microglia' related triangle occurrence numbers of cellular community in AD thirteen-month replicate 1 sample

Supplementary Data 26 P value of 'CTX-Ex' and 'Microglia' related node occurrence numbers of cellular community in AD thirteen-month replicate 2 sample

Supplementary Data 27 P value of 'CTX-Ex' and 'Microglia' related edge occurrence numbers of cellular community in AD thirteen-month replicate 2 sample

Supplementary Data 28 P value of 'CTX-Ex' and 'Microglia' related triangle occurrence numbers of cellular community in AD thirteen-month replicate 2 sample

Supplementary Data 29 Pathway retrieving results for differentiable values among CellChat matrices of motif 'CCC' in eight and thirteen-month samples

Supplementary Data 30 Pathway retrieving results for differentiable values among CellChat matrices of motif 'CCM' in eight and thirteen-month samples

Supplementary Data 31 Pathway retrieving results for differentiable values among CellChat matrices of motif 'CMM' in eight and thirteen-month samples

Supplementary Data 32 Pathway retrieving results for differentiable values among CellChat matrices of motif 'MMM' in eight and thirteen-month samples

Supplementary Data 33 Cell-cell Communication matrix of motif 'CCC' and related 3-hop regions in AD eight-month samples inferred from CellChat using TruncatedMean

Supplementary Data 34 Cell-cell Communication matrix of motif 'CCM' and related 3-hop regions in AD eight-month samples inferred from CellChat using TruncatedMean

Supplementary Data 35 Cell-cell Communication matrix of motif 'CMM' and related 3-hop regions in AD eight-month samples inferred from CellChat using TruncatedMean

Supplementary Data 36 Cell-cell Communication matrix of motif 'MMM' and related 3-hop regions in AD eight-month samples inferred from CellChat using TruncatedMean

Supplementary Data 37 Cell-cell Communication matrix of motif 'CCC' and related 3-hop regions in AD thirteen-month samples inferred from CellChat using TruncatedMean

Supplementary Data 38 Cell-cell Communication matrix of motif 'CCM' and related 3-hop regions in AD thirteen-month samples inferred from CellChat using TruncatedMean

Supplementary Data 39 Cell-cell Communication matrix of motif 'CMM' and related 3-hop regions in AD thirteen-month samples inferred from CellChat using TruncatedMean

Supplementary Data 40 Cell-cell Communication matrix of motif 'MMM' and related 3-hop regions in AD thirteen-month samples inferred from CellChat using TruncatedMean

Supplementary Data 41 Differentially Expressed Genes of Motif 'CCC' in AD eight-month samples using Wilcoxon test

Supplementary Data 42 Differentially Expressed Genes of Motif 'CCM' in AD eight-month samples using Wilcoxon test

Supplementary Data 43 Differentially Expressed Genes of Motif 'CMM' in AD eight-month samples using Wilcoxon test

Supplementary Data 44 Differentially Expressed Genes of Motif 'MMM' in AD eight-month samples using Wilcoxon test

Supplementary Data 45 Differentially Expressed Genes of Motif 'CCC' in AD thirteen-month samples using Wilcoxon test

Supplementary Data 46 Differentially Expressed Genes of Motif 'CCM' in AD thirteen-month samples using Wilcoxon test

Supplementary Data 47 Differentially Expressed Genes of Motif 'CMM' in AD thirteen-month samples using Wilcoxon test

Supplementary Data 48 Differentially Expressed Genes of Motif 'MMM' in AD thirteen-month samples using Wilcoxon test

Supplementary Data 49 Pathway retrieving results for differentiable values among CellChat matrices of motif 'CCCM' in eight and thirteen-month samples

Supplementary Data 50 Pathway retrieving results for differentiable values among CellChat matrices of motif 'MMMM' in eight and thirteen-month samples

Supplementary Data 51 Pathway retrieving results for differentiable values among CellChat matrices of motif 'MMMC' in eight and thirteen-month samples

Supplementary Data 52 Differentially Expressed Genes of Motif 'CCCM' in AD eight-month samples using Wilcoxon test

Supplementary Data 53 Differentially Expressed Genes of Motif 'MMMM' in AD eight-month samples using Wilcoxon test

Supplementary Data 54 Differentially Expressed Genes of Motif MMMC' in AD eight-month samples using Wilcoxon test

Supplementary Data 55 Differentially Expressed Genes of Motif 'CCCM' in AD thirteen-month samples using Wilcoxon test

Supplementary Data 56 Differentially Expressed Genes of Motif 'MMMM' in AD thirteen-month samples using Wilcoxon test

Supplementary Data 57 Differentially Expressed Genes of Motif MMMC' in AD thirteen-month samples using Wilcoxon test

Supplementary Data 58 Cell-cell Communication matrix of motif 'CCCM' and related 3-hop regions in AD eight-month samples inferred from CellChat using TruncatedMean

Supplementary Data 59 Cell-cell Communication matrix of motif 'MMMM' and related 3-hop regions in AD eight-month samples inferred from CellChat using TruncatedMean

Supplementary Data 60 Cell-cell Communication matrix of motif 'MMMC' and related 3-hop regions in AD eight-month samples inferred from CellChat using TruncatedMean

Supplementary Data 61 Cell-cell Communication matrix of motif 'CCCM' and related 3-hop regions in AD thirteen-month samples inferred from CellChat using TruncatedMean

Supplementary Data 62 Cell-cell Communication matrix of motif 'MMMM' and related 3-hop regions in AD thirteen-month samples inferred from CellChat using TruncatedMean

Supplementary Data 63 Cell-cell Communication matrix of motif 'MMMC' and related 3-hop regions in AD thirteen-month samples inferred from CellChat using TruncatedMean

Supplementary Data 64 P value of edges (size-2 CC motifs) occurrence numbers of cellular community in CRC MIBI-TOF dataset

Supplementary Data 65 P value of triangles (size-3 CC motifs) containing 'CD8 T-cells' and 'Epithelial' cell types (and all triangles) in CRC MIBI-TOF dataset

Supplementary Data 66 Proportion test on size-3 and size-4 CC motifs in AD case study

Supplementary Data 67 Occurrence numbers of size-3 and size-4 Shifted Interaction Motifs across different samples in colorectal carcinoma. CRC37_0092 are samples from patients at stage II, CRC35_0086 are samples from patients at stage III, and CRC38_0028 are samples from patients at stage IV

Supplementary Data 68 Occurrence numbers of size-3 and size-4 the Homeostatic Interaction Motif across different samples

Supplementary Data 69 top 300 significant GO terms in the intersection of four size-3 motifs: 'CCC', 'CCM', 'CMM', and 'MMM' in 13-month-old AD disease samples

Supplementary Data 70 all 821 significant GO terms in the intersection of four size-3 motifs: 'CCC', 'CCM', 'CMM', and 'MMM' in 13-month-old AD disease samples

Supplementary Data 71 all 27 significant pathways intersecting four size-3 motifs: 'CCC', 'CCM', 'CMM', and 'MMM' in 13-month-old AD disease samples

Supplementary Data 72 Occurrence numbers of size-3 and size-4 simulated CC motifs
